# Comparative Efficacy of Targeted Therapies in Patients with Non-Small Cell Lung Cancer: A Network Meta-Analysis of Clinical Trials

**DOI:** 10.3390/jcm9041063

**Published:** 2020-04-09

**Authors:** Tung Hoang, Seung-Kwon Myung, Thu Thi Pham, Jeongseon Kim, Woong Ju

**Affiliations:** 1 Department of Cancer Biomedical Science, National Cancer Center Graduate School of Cancer Science and Policy, Goyang 10408, Korea; 75256@ncc.re.kr (T.H.); jskim@ncc.re.kr (J.K.); 2 Division of Cancer Epidemiology and Management, National Cancer Center Research Institute, Goyang 10408, Korea; 3 Department of Family Medicine and Center for Cancer Prevention and Detection, National Cancer Center Hospital, Goyang 10408, Korea; 4 Health Data Science Program, Institute of Public Health, Charité Universitätsmedizin Berlin, 10117 Berlin, Germany; thuphamhup@gmail.com; 5 Molecular Epidemiology Research Group, Max Delbrück Center for Molecular Medicine (MDC), 13125 Berlin, Germany; 6 Department of Obstetrics and Gynecology, Ewha Womans University College of Medicine, Seoul 07804, Korea; goodmorning@ewha.ac.kr; 7 Medical Research Institute, Ewha Womans University College of Medicine, Seoul 07804, Korea

**Keywords:** non-small cell lung cancer, targeted therapy, network meta-analysis

## Abstract

This study aims to investigate the efficacy of targeted therapies in the treatment of non-small cell lung cancer (NSCLC) by using a network meta-analysis of clinical trials. PubMed, EMBASE, Cochrane Library, and Clinicaltrials.gov were searched by using keywords related to the topic on 19 September 2018. Two investigators independently selected relevant trials by pre-determined criteria. A pooled response ratio (RR) for overall response rate (ORR) and a hazard ratio (HR) for progression-free survival (PFS) were calculated based on both the Bayesian and frequentist approaches. A total of 128 clinical trials with 39,501 participants were included in the final analysis of 14 therapeutic groups. Compared with chemotherapy, both ORR and PFS were significantly improved for afatinib, alectinib, and crizotinib, while only PFS was significantly improved for cabozantinib, ceritinib, gefitinib, and osimertinib. Consistency was observed between the direct and indirect comparisons based on the Bayesian approach statistically and the frequentist approach visually. Cabozantinib and alectinib showed the highest probability for the first-line treatment ranking in ORR (62.5%) and PFS (87.5%), respectively. The current network meta-analysis showed the comprehensive evidence-based comparative efficacy of different types of targeted therapies, which would help clinicians use targeted therapies in clinical practice.

## 1. Introduction

Lung cancer is the most common cancer and the leading cause of cancer death worldwide, with approximately 2.1 million new cases (11.6% of the total new cases) and 1.76 million deaths (18.4% of the total deaths) [1,2]. Of the two major types of lung cancer, non-small cell lung cancer (NSCLC) accounts for about 85% to 90% of all lung cancers, which typically has a slower rate and double time than small cell lung cancer [3,4].

Among several treatment options for NSCLC treatment recommended by the latest updated National Comprehensive Cancer Network (NCCN) guideline, targeted cancer therapy with various pathways is one of the new generations of cancer treatments [5]. Some cell surface receptors such as epidermal growth factor receptor (EGFR), anaplastic lymphoma kinase (ALK), and receptor of silencing 1 (ROS1) are overactive in the pathology of NSCLC [6,7]. Also, B-Raf proto-oncogene (BRAF), kirsten rat sarcoma 2 viral oncogene homolog (KRAS) and a kinase upstream of mitogen-activated protein kinase (MEK) have generated recent interest [8]. Other inhibitors of human epidermal growth factor receptor 2 (HER2), ‘rearranged during transfection’ proto-oncogene (RET), and tyrosine-protein kinase Met (MET) have also been approved for the treatment of NSCLC [9,10,11]. Although the efficacy of targeted therapies has been evaluated through large-scale randomized controlled trials and has already been approved by the Food and Drug Administration (FDA), their comparative efficacy has not been investigated.

Therefore, we performed a network meta-analysis (NMA) of clinical trials to compare and rank targeted therapies for the treatment of patients with NSCLC.

## 2. Materials and Methods

### 2.1. Search Strategy and Keywords

Eligible studies were identified by searching PubMed, EMBASE, Cochrane library, and Clinicaltrials.gov databases from their inception until September 19, 2018, limiting to human subjects and a clinical trial. The keywords for literature search were as follows: ‘ado-trastuzumab’, ‘afatinib’, ‘alectinib’, ‘bevacizumab’, ‘brigatinib’, ‘cabozantinib’, ‘ceritinib’, ‘cetuximab’, ‘crizotinib’, ‘dabrafenib’, ‘erlotinib’, ‘gefitinib’, ‘osimertinib’, ‘ramucirumab’, ‘trametinib’, ‘vandetanib’, and ‘vemurafenib’ for intervention factors; ‘non-small cell lung cancer’ for an outcome factor; and ‘clinical trial’ and ‘randomized controlled trial’ for type of study. The bibliographies of relevant articles were also reviewed to identify additional studies related to this topic. The literature search was restricted to studies published in English.

### 2.2. Selection of Relevant Studies

We included head-to-head or controlled trials that: compared the efficacy of FDA-approved targeted drugs with chemotherapy or placebos in the treatment of NSCLC; reported the outcomes on overall response rates (ORRs) and/or hazard ratios (HRs) for progression-free survival (PFS).

Two investigators (Hoang and Myung) independently selected relevant trials searched from the databases. The following variables were extracted from all the included studies: study name (first author, published year, and specific trial title, if possible), period and country, regimen of the intervention and the comparison, number of participants, and main outcomes.

### 2.3. Data Analysis

The pooled response ratio (RR) for ORRs based on an arm-based approach, HR for PFS based on a contrast-based approach, and their 95% confidence intervals (95% CIs) were calculated for estimating the differences between treatment groups.

We measured inconsistency, which implies statistical disagreement between direct and indirect comparisons [12,13]. The generalized linear model was applied for the Bayesian NMA [14]. Binomial likelihood and logit link function were applied for arm-based data of ORR, while normal likelihood and identity link function were used for contrast-based data of natural logarithm HR in the Bayesian approach [14]. Also, Bayesian model assumptions in the Bayesian analysis were assessed by the convergence diagnostics of the Markov chain Monte Carlo [14].

Based on the ranking probabilities of each therapy in different treatment lines, we calculated the surface under the cumulative ranking line (SUCRA) value and performed k-means clustering analysis to group the similar treatments [15,16].

For the statistical analysis of this NMA, we used different packages including pcnetmeta, gemtc, and netmeta in the R statistical environment [17,18,19]. Results from both the Bayesian approach (pcnetmeta and gemtc packages) and the frequentist approach (netmeta package) and were presented.

Finally, we calculated a decremental hazard-response ratio (DHRR) to obtain a decreased amount of HR per a unit of RR (compared to a dummy group) as in the following formula:

$$\mathrm{DHRR}=-\frac{\mathrm{HR}-{\mathrm{HR}}_{o}}{\mathrm{RR}-{\mathrm{RR}}_{o}}$$

where ${\mathrm{HR}}_{o}$ and ${\mathrm{RR}}_{o}$ are a baseline hazard ratio and a response ratio of chemotherapy vs. a dummy group, respectively.

## 3. Results

### 3.1. Selection of Relevant Studies

Figure S1 shows the flow diagram for selection of relevant studies. We identified 7279 articles from four different databases (PubMed, EMBASE, Cochrane Library, and Clinicaltrials.gov) using the keywords and hand-search from relevant bibliographies. After excluding 845 duplicated records and 5815 irrelevant studies, the full text of the remaining 619 articles were reviewed. Overall, a total of 128 parallel clinical trials were included in the current network meta-analysis.

### 3.2. Study Characteristics

The general characteristics of the included studies (eReferences in the Supplement) were summarized in Table S1. A total of 39,501 study participants were assigned to receive 14 different treatments including 12 targeted therapies, 1 chemotherapy, and 1 dummy. Sixty-four % of all the studies involved the comparisons between EGFR-targeted drugs and other treatments.

### 3.3. Network Geometry

**Figure 1:**
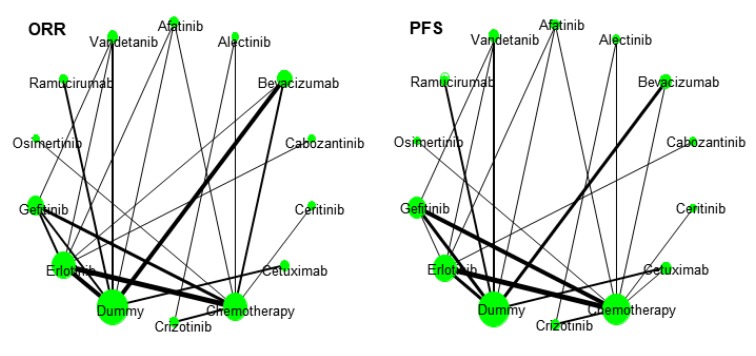
Network geometry of comparisons for overall response rate (ORR) and progression-free survival (PFS).

Figure 1 shows the network geometry for ORR and PFS to represent graphical comparisons among various treatments. The comparative efficacy between erlotinib vs. chemotherapy/bevacizumab vs. dummy/erlotinib vs. dummy was frequently investigated for ORR, while the comparative efficacy between erlotinib vs. chemotherapy/gefitinib vs. chemotherapy/gefitinib vs. dummy/bevacizumab vs. dummy/erlotinib vs. dummy was done for PFS.

### 3.4. Assumption Checking

Figures S2 and S3 show a heat map, which provides visual inconsistency between direct and indirect comparisons in the frequentist approach. There was a big difference between inconsistency before and after the detachment in some treatment comparisons. However, no inconsistency was observed in the Bayesian approach (Figures S4 and S5).

Substantial heterogeneity was detected in both ORR and PFS, with the global I^2^ = 78% for both outcomes as well as for either a pairwise pooled effect or a consistency effect (Table S2).

The width of every line reflects the number of studies. The size of the circles is proportional to the number of study participants. A dummy group is a placebo or a control group without additional treatment.

### 3.5. Comparative Efficacy

**Figure 2:**
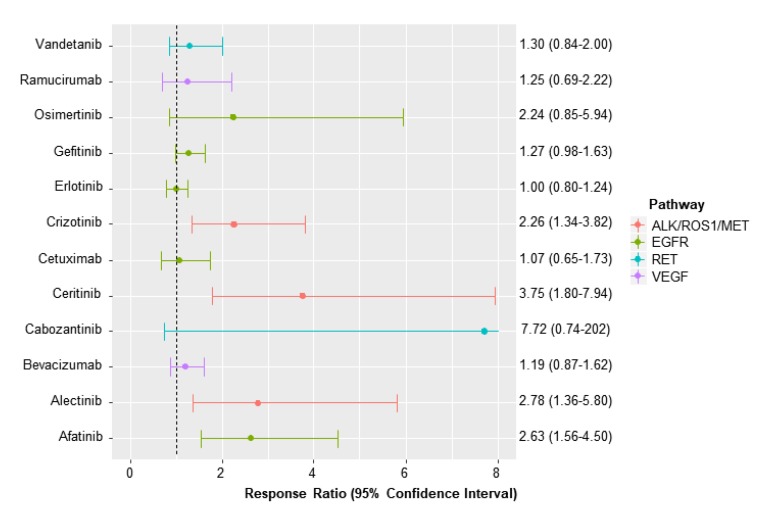
Response ratio for overall response rate of each targeted therapy vs. chemotherapy.

**Figure 3:**
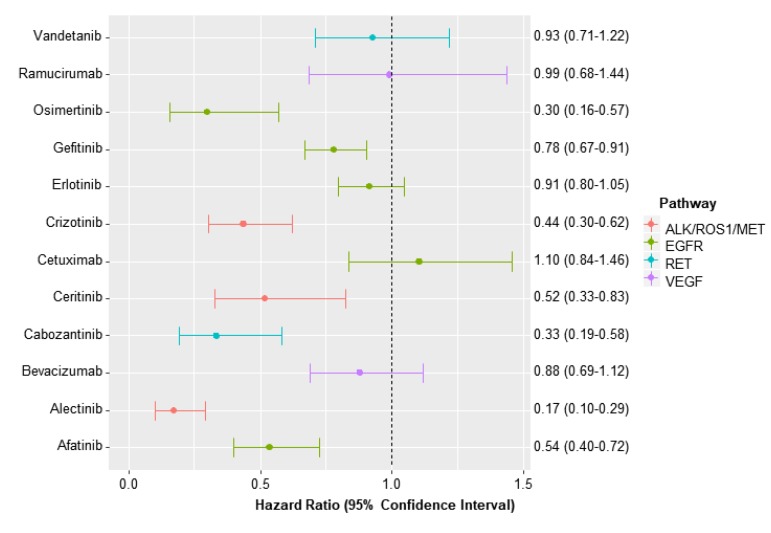
Hazard ratio for progression-free survival of each targeted therapy vs. chemotherapy.

Compared to chemotherapy, afatinib, alectinib, ceritinib, and crizotinib were found to have a higher ORR with RRs ranging between 2.26 (95% CI, 1.34–3.82) for crizotinib and 3.75 (95% CI, 1.80–7.94) for ceritinib (Figure 2). Also, cabozantinib, gefitinib, and osimertinib vs. chemotherapy were found to improve PFS with HRs ranging from 0.17 (95% CI, 0.10–0.29) for alectinib to 0.78 (0.67–0.91) for gefitinib (Figure 3).

Table 1 and Table 2 show the league tables representing the comparative efficacy of targeted therapies for ORR and PFS in the network meta-analysis based on the Bayesian approach.

Among EGFR inhibitors, ORR was found to be significantly higher in afatinib treatment, compared to cetuximab (RR, 2.46; 95% CI, 1.25–4.90), erlotinib (RR, 2.64; 95% CI, 1.54–4.58), and gefitinib (RR, 2.08; 95% CI, 1.18-3.68) (Table 1). Also, afatinib had a significantly longer PFS, compared to cetuximab (HR, 0.49; 95% CI, 0.33–0.71), erlotinib (HR, 0.59; 95% CI 0.44–0.80), and gefitinib (HR, 0.69; 95% CI, 0.50–0.95) (Table 2). Osimertinib was found to improve PFS, compared to cetuximab (HR, 0.27; 95% CI, 0.14–0.55), erlotinib (HR, 0.33; 95% CI, 0.17–0.64), and gefitinib (HR, 0.39; 95% CI, 0.20–0.75) (Table 2). Gefitinib showed a better PFS compared to cetuximab (HR, 0.70; 95% CI, 0.53–0.94) (Table 2).

Regarding ALK/ROS1/MET targeted drugs, there were no significant differences in ORR between each pair of crizotinib, ceritinib, and alectinib (Table 1). However, alectinib showed a superior efficacy compared to either crizotinib (HR, 0.40; 95% CI, 0.25–0.64) or ceritinib (HR, 0.33; 0.17–0.67) for PFS (Table 2).

As for VEGF pathway (bevacizumab and ramucizumab) and RET targeted therapy (cabozantinib and vandetanib), only cabozantinib was found to improve PFS compared to vandetanib (HR, 0.36; 95% CI, 0.20–0.66) (Table 2).

### 3.6. Sensitivity Analysis

Findings of the direct pairwise meta-analysis and the relative effect estimates for ORR and PFS using the frequentist approach are presented in Tables S3–S5. The findings were similar to those by using the Bayesian approach (Table 1 and Table 2).

### 3.7. Treatment Ranking

The Gelman plot for checking Bayesian model assumption shows a low chain reduction over time for both ORR and PFS outcomes, and the chains seem roughly converged after maximum 10,000 iterations in chain (Figures S6 and S7). Also, cabozantinib and alectinib were found to become the first-line therapies with the highest treatment ranking probabilities of 62.5% for ORR and 87.5% for PFS, respectively (Tables S6 and S7 and Figure S8). In the k-means clustering analysis of SUCRA, ceritinib, alectinib, crizotinib, osimertinib, cabozantinib, and afatinib showed the more efficacy compared with the remaining treatment (Figure S9).

Figure S10 reports the two-dimensional graphs about RR for ORR and HR for PFS in the comparison with dummy group. DHRR indicated the decrease of HR obtained per 1 unit increase of RR for osimertinib (0.34), alectinib (0.28), bevacizumab (0.38), and vandetanib (0.14), which are higher than that for other drugs relating to EGFR, ALK/ROS1/MET, VEGF, and RET pathways.

## 4. Discussion

### 4.1. Summary of Findings

In the current comprehensive network meta-analysis, compared to chemotherapy, most of the targeted drugs including afatinib, alectinib, cabozantinib, ceritinib, crizotinib, gefitinib, and osimertinib showed a significantly higher efficacy in ORR and PFS. Among EGFR inhibitors, afatinib was found to improve both ORR and PFS, vs. cetuximab, erlotinib, or gefitinib treatment. Furthermore, alectinib and cabozantinib also showed the lower risk of disease progression, compared to other drugs in the ALK/ROS1/MET and RET pathways.

There was no inconsistency between direct and indirect comparisons in most treatments based on the Bayesian approach. The findings of the NMA based on both the frequentist and Bayesian approach were similar in pooled effect sizes as well as a significant direction. Also, Bayesian assumptions were ensured by convergence diagnostics.

### 4.2. Comparison with Previous Studies

Previous reports related to EGFR inhibitors showed consistent findings with the current study. A recent meta-analysis of 90 retrospective or prospective cohort studies and clinical trials showed the comparable effect of gefitinib vs. erlotinib [20]. The RR (95% CI) for ORR and HR (95% CI) for PFS were 1.05 (1.00–1.11) and 1.00 (0.95–1.04), respectively [20]. Another network meta-analysis of 11 clinical trials also showed the similar PFS between gefitinib and erlotinib [21]. However, unlike our findings, the third-generation EGFR inhibitor osimertinib was found to have a longer PFS (HR 0.71, 95% CI 0.54–0.95), and the significant difference between the second-generation EGFR inhibitor afatinib and standard of care (either gefitinib or erlotinib) was not observed (HR 0.96, 95% CI, 0.86–1.17) [21].

In a large medical chart review of 1471 participants with ALK-positive NSCLC among a total of 27,375 recorded subjects from seven countries, crizotinib showed a significant improvement in complete response (odds ratio (OR) = 2.65, 95% CI = 1.69–4.15) and reduction of recurrence/progression (odds ratio = 0.38, 95% CI = 0.24–0.59) compared to controls [22]. Also, a recent network meta-analysis of ALK inhibitors showed consistent findings among treatments in both ORR and PFS outcomes [23]. In Fan et al.’s study, a remarkable improvement in ORR was shown: the ORs (95%CI) for crizotinib, ceritinib, and alextinib were 11.69 (4.29–36.56), 7.85 (3.44–19.27), and 6.04 (3.33–11.71), compared to chemotherapy, respectively [23]. The superior efficacy of alectinib in PFS might be associated with the resistance to crizotinib among ALK-positive NSCLC patients, which reduces therapeutic response to crizotinib [24,25]. Although ceritinib is also a second-generation ALK inhibitor, our study showed that there is no signicant difference in the efficacy between ceritinib and crizotinib. Similarly, the recent meta-analyses of pooled estimates reported that crizotinib might have higher ORR [66% (58%–74%) vs. 52% (38%–66%)] and longer PFS [9.27 months (8.28–10.26) vs. 5.92 months (4.36–7.48)] than ceritinib, although no statistical test was performed [26]. It remains unclear why ceritinib did not show a superior efficacy unlike alectinib.

### 4.3. Strengths and Limitations

To the best of our knowledge, this is the first network meta-analysis which summarized the direct and indirect evidence on the comparative efficacy of targeted therapies in the treatment of NSCLC. Also, this compiled a large dataset, and the method was valid by checking several assumptions. In addition, this network meta-analysis included clinical trials only, which had a higher level of evidence than observational studies and allowed us to obtain the precise estimates.

Despite the strengths, there are several limitations in the current study. The efficacy of targeted therapies was evaluated through ORR and PFS surrogates only. We did not perform subgroup analyses by different treatment lines and patients of different mutations as well. Also, the potential heterogeneity was observed with approximately 78% for both ORR and PFS outcomes. Finally, among 34,969 subjects included for the analysis of ORR outcome, the small number of patients received cabozatinib (38 subjects, Table S1). Also, a big difference in ORRs between the two arms (10.5% for cabozatinib vs. 2.6% erlotinib) might lead to the large error margins for the comparative effect of cabozantinib and other treatments (Figure 2 and Table 1).

## 5. Conclusions

In summary, the current study showed the comprehensive evidence-based comparative efficacy of different types of targeted therapies, which would help clinicians use targeted therapies in clinical practice. Cabozantinib and alectinib showed the highest probability for the first-line treatment ranking in ORR and PFS, respectively.

## Figures and Tables

**Table 1:** Comparative efficacy of targeted therapies for overall response rate in the network meta-analysis based on the Bayesian approach.

Afat	0.95 (0.38–2.31)	*2.22 (1.25–3.98)*	0.34 (0.01–3.81)	0.71 (0.28–1.73)	*2.46 (1.25–4.90)*	*2.63 (1.56–4.50)*	1.18 (0.55–2.46)	*3.53 (2.06–6.15)*	*2.64 (1.54–4.58)*	*2.08 (1.18–3.68)*	1.18 (0.39–3.51)	2.11 (1.00–4.49)	*2.03 (1.07–3.88)*
1.06 (0.43–2.62)	Alec	*2.34 (1.08–5.22)*	0.36 (0.01–4.26)	0.74 (0.26–2.10)	*2.60 (1.10–6.27)*	*2.78 (1.36–5.80)*	1.24 (0.67–2.27)	*3.74 (1.75–8.22)*	*2.79 (1.32–6.02)*	*2.20 (1.03–4.78)*	1.24 (0.37–4.21)	2.24 (0.90–5.71)	2.15 (0.93–5.03)
*0.45 (0.25–0.80)*	*0.42 (0.19–0.93)*	Beva	0.15 (0.01–1.63)	*0.31 (0.14–0.70)*	1.11 (0.70–1.78)	1.19 (0.87–1.62)	*0.52 (0.28–0.96)*	*1.59 (1.25–2.05)*	1.19 (0.87–1.63)	0.94 (0.66–1.32)	0.53 (0.19–1.46)	0.95 (0.54–1.69)	0.92 (0.59–1.41)
2.93 (0.26–78.11)	2.79 (0.23–76.6)	6.49 (0.62–170)	Cabo	2.08 (0.17–57.3)	7.21 (0.67–193)	7.72 (0.74–202)	3.45 (0.30–92.0)	10.36 (0.99–272)	7.74 (0.75–201)	6.09 (0.58–162)	3.45 (0.27–104)	6.20 (0.56–166)	5.95 (0.56–157)
1.43 (0.58–3.57)	1.35 (0.48–3.78)	*3.16 (1.43–7.12)*	0.49 (0.02–5.78)	Ceri	*3.51 (1.45–8.60)*	*3.75 (1.80–7.94)*	1.66 (0.68–4.13)	*5.04 (2.30–11.2)*	*3.76 (1.74–8.25)*	*2.96 (1.36–6.54)*	1.68 (0.50–5.65)	*3.02 (1.18–7.83)*	*2.89 (1.23–6.89)*
*0.41 (0.20–0.80)*	*0.38 (0.16–0.91)*	0.90 (0.56–1.44)	0.14 (0.01–1.49)	*0.28 (0.12–0.69)*	Cetu	1.07 (0.65–1.73)	0.47 (0.23–0.96)	1.44 (0.96–2.14)	1.07 (0.67–1.72)	0.84 (0.51–1.38)	0.48 (0.16–1.41)	0.86 (0.45–1.65)	0.82 (0.48–1.41)
*0.38 (0.22–0.64)*	*0.36 (0.17–0.74)*	0.84 (0.62–1.15)	0.13 (0.00–1.35)	*0.27 (0.13–0.56)*	0.93 (0.58–1.53)	Chem	*0.44 (0.26–0.75)*	*1.34 (1.03–1.77)*	1.00 (0.80–1.25)	0.79 (0.61–1.02)	0.45 (0.17–1.17)	0.80 (0.45–1.45)	0.77 (0.50–1.19)
0.86 (0.41–1.82)	0.81 (0.44–1.49)	*1.90 (1.04–3.51)*	0.29 (0.01–3.28)	0.60 (0.24–1.48)	*2.11 (1.04–4.34)*	*2.26 (1.34–3.82)*	Criz	*3.03 (1.69–5.50)*	*2.26 (1.29–4.03)*	1.78 (0.99–3.20)	1.01 (0.34–3.03)	1.81 (0.84–3.99)	1.74 (0.89–3.45)
*0.28 (0.16–0.49)*	*0.26 (0.12–0.57)*	*0.63 (0.49–0.80)*	0.10 (0.00–1.01)	*0.20 (0.09–0.43)*	0.70 (0.47–1.04)	*0.75 (0.57–0.98)*	*0.33 (0.18–0.59)*	Dum	*0.75 (0.58–0.96)*	*0.59 (0.44–0.78)*	*0.33 (0.12–0.91)*	0.60 (0.36–1.00)	*0.57 (0.40–0.82)*
*0.38 (0.22–0.65)*	*0.36 (0.17–0.76)*	0.84 (0.61–1.15)	0.13 (0.00–1.33)	*0.26 (0.12–0.57)*	0.93 (0.58–1.50)	1.00 (0.80–1.24)	*0.44 (0.25–0.78)*	*1.34 (1.05–1.72)*	Erlo	0.79 (0.59–1.04)	0.45 (0.16–1.19)	0.80 (0.46–1.42)	0.77 (0.51–1.16)
*0.48 (0.27–0.85)*	*0.45 (0.21–0.98)*	1.07 (0.76–1.51)	0.16 (0.01–1.72)	*0.34 (0.15–0.74)*	1.19 (0.73–1.94)	1.27 (0.98–1.63)	0.56 (0.31–1.01)	*1.70 (1.28–2.27)*	1.27 (0.96–1.68)	Gefi	0.57 (0.21–1.53)	1.02 (0.57–1.83)	0.98 (0.63–1.51)
0.85 (0.29–2.56)	0.80 (0.24–2.70)	1.88 (0.68–5.22)	0.29 (0.01–3.75)	0.59 (0.18–2.01)	2.09 (0.71–6.26)	2.24 (0.85–5.94)	0.99 (0.33–2.97)	*3.01 (1.10–8.30)*	2.24 (0.84–6.07)	1.77 (0.65–4.84)	Osim	1.80 (0.59–5.63)	1.73 (0.60–5.02)
0.47 (0.22–1.00)	0.44 (0.18–1.12)	1.05 (0.59–1.85)	0.16 (0.01–1.79)	*0.33 (0.13–0.84)*	1.16 (0.61–2.23)	1.25 (0.69–2.22)	0.55 (0.25–1.19)	*1.67 (1.00–2.78)*	1.25 (0.71–2.20)	0.98 (0.55–1.76)	0.56 (0.18–1.70)	Ramu	0.96 (0.51–1.79)
*0.49 (0.26–0.94)*	0.46 (0.20–1.07)	1.09 (0.71–1.69)	0.17 (0.01–1.77)	*0.34 (0.15–0.81)*	1.21 (0.71–2.09)	1.30 (0.84–2.00)	0.57 (0.29–1.13)	*1.74 (1.22–2.51)*	1.30 (0.86–1.97)	1.02 (0.66–1.59)	0.58 (0.20–1.67)	1.04 (0.56–1.96)	Vand

Drugs are reported in alphabetical order. Data in the right-upper triangle are RRs (95% confidence interval, CI) in the row-defining treatment compared with the column-defining treatment. RRs higher than 1 favor the row-defining treatment (the first drug in alphabetical order). RRs for the opposite comparison of ORR are in the left-lower triangle. Each comparison is shown twice in the table, once with drug A vs. drug B and once with drug B vs. drug A. Significant results are in italic and underscored. RR, response ratio; CI, confidence interval; ORR: overall response rate; Afat, afatinib; Alec, alectinib; Beva, bevacizumab; Cabo, cabozantinib; Ceri, ceritinib; Cetu, cetuximab; Chem, chemotherapy; Criz, crizotinib; Dum, dummy; Erlo, erlotinib; Gefi, gefitinib; Osim, osimertinib; Ramu, ramucirumab; Vand, vandetanib.

**Table 2:** Comparative efficacy of targeted therapies for progression-free survival in the network meta-analysis based on the Bayesian approach.

Afat	*3.10 (1.69–5.65)*	*0.61 (0.43–0.87)*	1.61 (0.87–2.98)	1.03 (0.60–1.79)	*0.49 (0.33–0.71)*	*0.54 (0.40–0.72)*	1.23 (0.78–1.96)	*0.43 (0.32–0.58)*	*0.59 (0.44–0.80)*	*0.69 (0.50–0.95)*	1.79 (0.88–3.63)	*0.54 (0.35–0.84)*	*0.58 (0.40–0.84)*
*0.32 (0.18–0.59)*	Alec	*0.20 (0.11–0.35)*	0.52 (0.24–1.11)	*0.33 (0.17–0.67)*	*0.16 (0.09–0.28)*	*0.17 (0.10–0.29)*	*0.40 (0.25–0.64)*	*0.14 (0.08–0.24)*	*0.19 (0.11–0.33)*	*0.22 (0.13–0.38)*	0.58 (0.25–1.33)	*0.17 (0.09–0.33)*	*0.19 (0.10–0.33)*
*1.64 (1.15–2.33)*	*5.08 (2.87–9.04)*	Beva	*2.63 (1.46–4.76)*	*1.69 (1.01–2.86)*	0.80 (0.59–1.08)	0.88 (0.69–1.12)	*2.02 (1.32–3.10)*	*0.71 (0.58–0.86)*	0.96 (0.76–1.23)	1.13 (0.88–1.45)	*2.93 (1.47–5.82)*	0.89 (0.60–1.30)	0.95 (0.70–1.28)
0.62 (0.34–1.15)	1.93 (0.90–4.12)	*0.38 (0.21–0.69)*	Cabo	0.64 (0.31–1.32)	*0.30 (0.16–0.55)*	*0.33 (0.19–0.58)*	0.77 (0.40–1.49)	*0.27 (0.15–0.47)*	*0.37 (0.21–0.63)*	*0.43 (0.24–0.76)*	1.11 (0.48–2.60)	*0.34 (0.18–0.65)*	*0.36 (0.20–0.66)*
0.97 (0.56–1.67)	*3.00 (1.50–6.02)*	*0.59 (0.35–0.99)*	1.55 (0.76–3.20)	Ceri	*0.47 (0.27–0.81)*	*0.52 (0.33–0.83)*	1.19 (0.67–2.14)	*0.42 (0.26–0.68)*	*0.57 (0.35–0.92)*	0.67 (0.41–1.09)	1.73 (0.78–3.85)	*0.52 (0.29–0.95)*	*0.56 (0.33–0.96)*
*2.05 (1.41–2.99)*	*6.37 (3.54–11.5)*	1.26 (0.92–1.71)	*3.30 (1.80–6.07)*	*2.12 (1.24–3.64)*	Cetu	1.10 (0.84–1.46)	*2.54 (1.62–3.98)*	0.89 (0.70–1.13)	1.21 (0.91–1.59)	*1.42 (1.06–1.89)*	*3.68 (1.83–7.39)*	1.11 (0.74–1.67)	1.19 (0.85–1.66)
*1.86 (1.38–2.51)*	*5.77 (3.42–9.73)*	1.14 (0.89–1.44)	*2.99 (1.72–5.21)*	*1.92 (1.21–3.06)*	0.91 (0.69–1.20)	Chem	*2.30 (1.61–3.29)*	*0.81 (0.68–0.95)*	1.09 (0.96–1.25)	*1.29 (1.10–1.50)*	*3.33 (1.75–6.34)*	1.01 (0.70–1.46)	1.08 (0.82–1.41)
0.81 (0.51–1.29)	*2.51 (1.57–4.00)*	*0.50 (0.32–0.76)*	1.30 (0.67–2.52)	0.84 (0.47–1.50)	*0.39 (0.25–0.62)*	*0.44 (0.30–0.62)*	Criz	*0.35 (0.24–0.52)*	*0.48 (0.33–0.70)*	*0.56 (0.38–0.83)*	1.45 (0.69–3.04)	*0.44 (0.26–0.73)*	*0.47 (0.30–0.73)*
*2.30 (1.71–3.11)*	*7.15 (4.14–12.4)*	*1.41 (1.16–1.71)*	*3.71 (2.12–6.49)*	*2.38 (1.46–3.91)*	1.12 (0.88–1.43)	*1.24 (1.05–1.46)*	*2.85 (1.92–4.22)*	Dum	*1.36 (1.16–1.59)*	*1.59 (1.34–1.89)*	*4.13 (2.12–8.02)*	1.25 (0.90–1.73)	*1.33 (1.05–1.68)*
*1.70 (1.26–2.30)*	*5.27 (3.07–9.07)*	1.04 (0.82–1.32)	*2.73 (1.60–4.68)*	*1.76 (1.09–2.85)*	0.83 (0.63–1.09)	0.91 (0.80–1.05)	*2.10 (1.44–3.07)*	*0.74 (0.63–0.86)*	Erlo	1.17 (0.98–1.40)	*3.05 (1.57–5.88)*	0.92 (0.64–1.33)	0.98 (0.76–1.28)
*1.45 (1.05–1.99)*	*4.49 (2.62–7.75)*	0.88 (0.69–1.13)	*2.33 (1.32–4.10)*	1.50 (0.92–2.44)	*0.70 (0.53–0.94)*	*0.78 (0.67–0.91)*	*1.79 (1.21–2.63)*	*0.63 (0.53–0.74)*	0.85 (0.71–1.02)	Gefi	*2.59 (1.33–5.03)*	0.78 (0.54–1.14)	0.84 (0.64–1.10)
0.56 (0.28–1.14)	1.73 (0.75–3.97)	*0.34 (0.17–0.68)*	0.90 (0.38–2.10)	0.58 (0.26–1.28)	*0.27 (0.14–0.55)*	*0.30 (0.16–0.57)*	0.69 (0.33–1.44)	*0.24 (0.12–0.47)*	*0.33 (0.17–0.64)*	*0.39 (0.20–0.75)*	Osim	*0.30 (0.14–0.64)*	*0.32 (0.16–0.65)*
*1.84 (1.18–2.89)*	*5.72 (3.02–10.9)*	1.13 (0.77–1.66)	*2.97 (1.55–5.68)*	*1.91 (1.06–3.47)*	0.90 (0.60–1.36)	0.99 (0.68–1.44)	*2.28 (1.37–3.82)*	0.80 (0.58–1.12)	1.09 (0.75–1.56)	1.27 (0.88–1.85)	*3.31 (1.56–6.94)*	Ramu	1.07 (0.71–1.61)
*1.73 (1.19–2.50)*	*5.36 (2.99–9.67)*	1.06 (0.78–1.43)	*2.78 (1.53–5.06)*	*1.79 (1.04–3.06)*	0.84 (0.60–1.17)	0.93 (0.71–1.22)	*2.13 (1.36–3.33)*	*0.75 (0.59–0.95)*	1.02 (0.78–1.32)	1.19 (0.91–1.57)	*3.10 (1.54–6.23)*	0.94 (0.62–1.40)	Vand

Drugs are reported in alphabetical order. Data in the right-upper triangle are HRs (95% CI) in the row-defining treatment compared with the column-defining treatment. HRs lower than 1 favour the row-defining treatment (the first drug in alphabetical order). HRs for the opposite comparison of PFS are in the left-lower triangle. Each comparison is shown twice in the table, once with drug A vs. drug B and once with drug B vs. drug A. Significant results are in italic and underscored. HR, hazard ratio; CI, confidence interval; PFS: progression-free survival. Afat, afatinib; Alec, alectinib;. Beva, bevacizumab; Cabo, cabozantinib; Ceri, ceritinib; Cetu, cetuximab; Chem, chemotherapy;. Criz, crizotinib; Dum, dummy; Erlo, erlotinib; Gefi, gefitinib; Osim, osimertinib; Ramu, ramucirumab; Vand, vandetanib.

## Supplementary Materials

The following are available online at https://www.mdpi.com/2077-0383/9/4/1063/s1: Figure S1: Flow diagram for selection of relevant studies, Figure S2: Inconsistency-detecting heat map between direct and indirect comparisons for overall response rate in the frequentist approach, Figure S3: Inconsistency-detecting heat map between direct and indirect comparisons for progression-free survival in the frequentist approach, Figure S4: Node-splitting analysis of inconsistency for the comparison of overall response rate in the Bayesian approach, Figure S5: Node-splitting analysis of inconsistency for the comparison of progression-free survival in the Bayesian approach, Figure S6: Convergence diagnostics for the comparison of overall response rate, Figure S7: Convergence diagnostics for the comparison of progression-free survival, Figure S8: Treatment ranking plots according to overall response rate and progression-free survival, Figure S9: Cluster ranking plot based on SUCRA values of the overall response rate and progression free-survival of treatments, Figure S10: Two-dimensional graphs for response ratio and hazard ratio compared to dummy group, Table S1: General characteristics of the studies included in the final analysis, Table S2: Test for heterogeneity, Table S3: Direct pairwise comparative efficacy in the frequentist approach, Table S4: Comparative efficacy of targeted therapies for overall response rate in the network meta-analysis based on the frequentist approach, Table S5: Comparative efficacy of targeted therapies for progression-free survival in the network meta-analysis based on the frequentist approach, Table S6: Treatment ranking probability based on overall response rate, Table S7: Treatment ranking probability based on progression-free survival, eReferences: List of studies included in the network meta-analysis.
