# Supplementary material for: Comparative Efficacy of Targeted Therapies in Patients with Non-Small Cell Lung Cancer: A Network Meta-Analysis of Clinical Trials
Source: J Clin Med. 2020 Apr 9;9(4):1063. doi: 10.3390/jcm9041063 (PMC7230601; doi:10.3390/jcm9041063)
Supplement: Supplementary file 1 [file jcm-09-01063-s001.pdf]

SUPPLEMENTARY

Comparative Efficacy of Targeted Therapies in Patients with Non-Small Cell Lung Cancer: A Network Meta-Analysis of Clinical Trial

Figure S1. Flow diagram for selection of relevant studies

Figure S2. Inconsistency-detecting heat map between direct and indirect comparisons for overall response rate in the frequentist approach

Figure S3. Inconsistency-detecting heat map between direct and indirect comparisons for progression-free survival in the frequentist approach

Figure S4. Node-splitting analysis of inconsistency for the comparison of overall response rate in the Bayesian approach

Figure S5. Node-splitting analysis of inconsistency for the comparison of progression-free survival in the Bayesian approach

Figure S6. Convergence diagnostics for the comparison of overall response rate

Figure S7. Convergence diagnostics for the comparison of progression-free survival

Figure S8. Treatment ranking plots according to overall response rate and progression-free survival

Figure S9. Cluster ranking plot based on SUCRA values of the overall response rate and progression free-survival of treatments

Figure S10. Two-dimensional graphs for response ratio and hazard ratio compared to dummy group

Table S1. General characteristics of the studies included in the final analysis

Table S2. Test for heterogeneity

Table S3. Direct pairwise comparative efficacy in the frequentist approach

Table S4. Comparative efficacy of targeted therapies for overall response rate in the network meta-analysis based on the frequentist approach

Table S5. Comparative efficacy of targeted therapies for progression-free survival in the network meta-analysis based on the frequentist approach

Table S6. Treatment ranking probability based on overall response rate

Table S7. Treatment ranking probability based on progression-free survival

eReference. List of studies included in the network meta-analysis

Figure S1. Flow diagram for selection of relevant studies

Identified studies from databases by using keywords, filters, and bibliographies of relevant articles (N = 7,279):
PubMed (N = 1,087), EMBASE (N = 4,779), Cochrane Library (N = 1,127), Clinicaltrials.gov (N = 285), and bibliographies (N = 1)

→ Duplicate articles excluded (N = 845)

Articles with titles and abstracts reviewed (N = 6,434)

→ Excluded not relevant articles (N = 5,815)

Articles with full texts reviewed (N = 619)

→ Excluded articles not meeting the selection criteria (N = 491)
- No sufficient data (N = 47)
- Not relevant studies (N = 43)
- Not head-to-head neither controlled trials (N = 303): single arm trials (N = 99), phase 1 trials (N = 30), retrospective design (N = 21), and other (N = 153)
- Overlapping data (N = 78)
- Not orginal reports (N = 20): Review articles (N = 3), pooled analysis (N = 3), protocol (N = 14)

Clinical trials included in the final analysis (N = 128)

Figure S2. Inconsistency-detecting heat map between direct and indirect comparisons for overall response rate in the frequentist approach

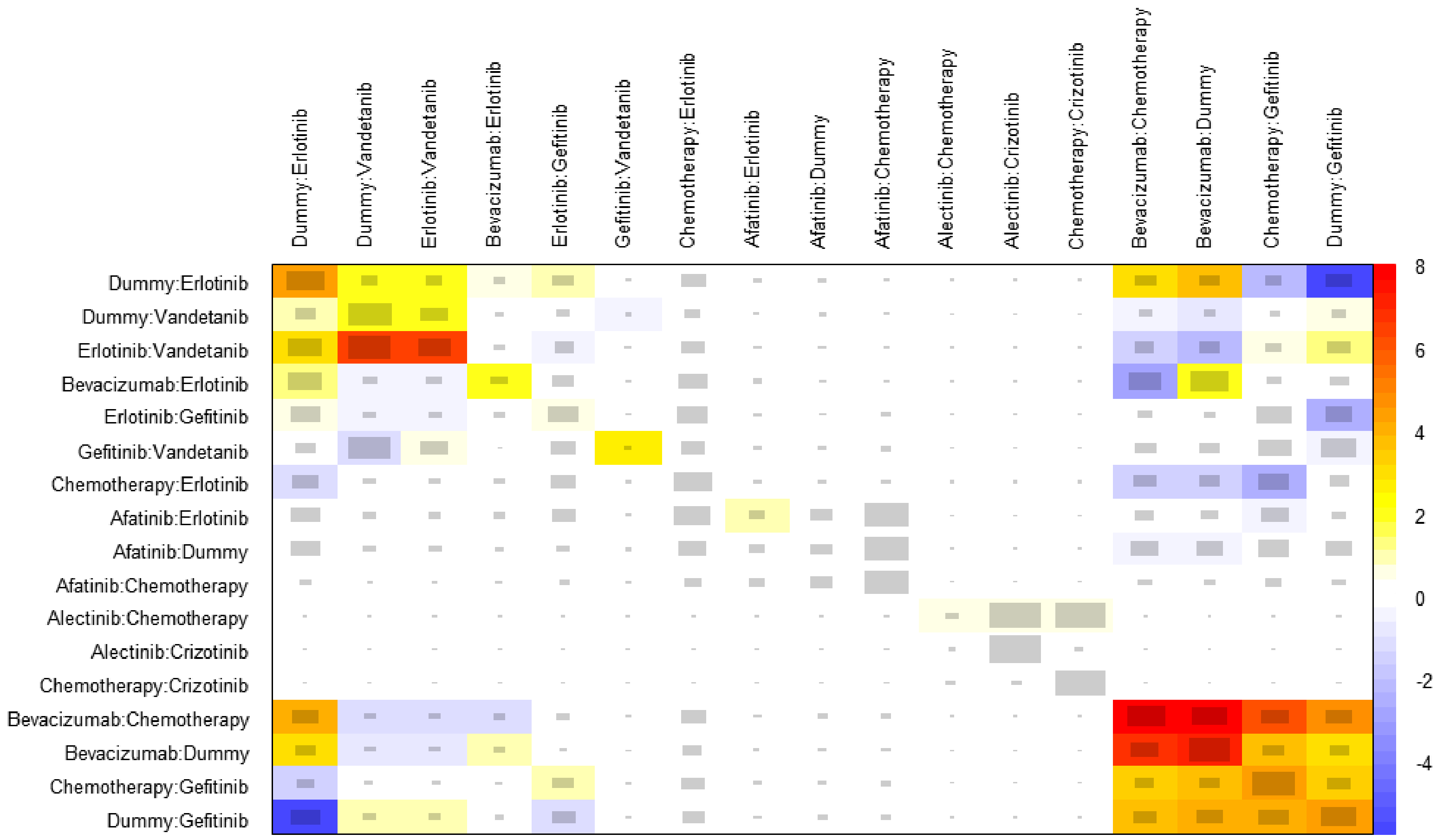

The gray square represents the contribution of the direct estimate of the column-defining comparison to the network estimate in the row. The colors on the diagonal display the inconsistency contribution of the corresponding comparison. The colors on the off-diagonal are related to the change in inconsistency between direct and indirect estimate in a network estimate in the row after relaxing the consistency assumption for the effect of one comparison in the column.

Figure S3. Inconsistency-detecting heat map between direct and indirect comparisons for progression-free survival in the frequentist approach

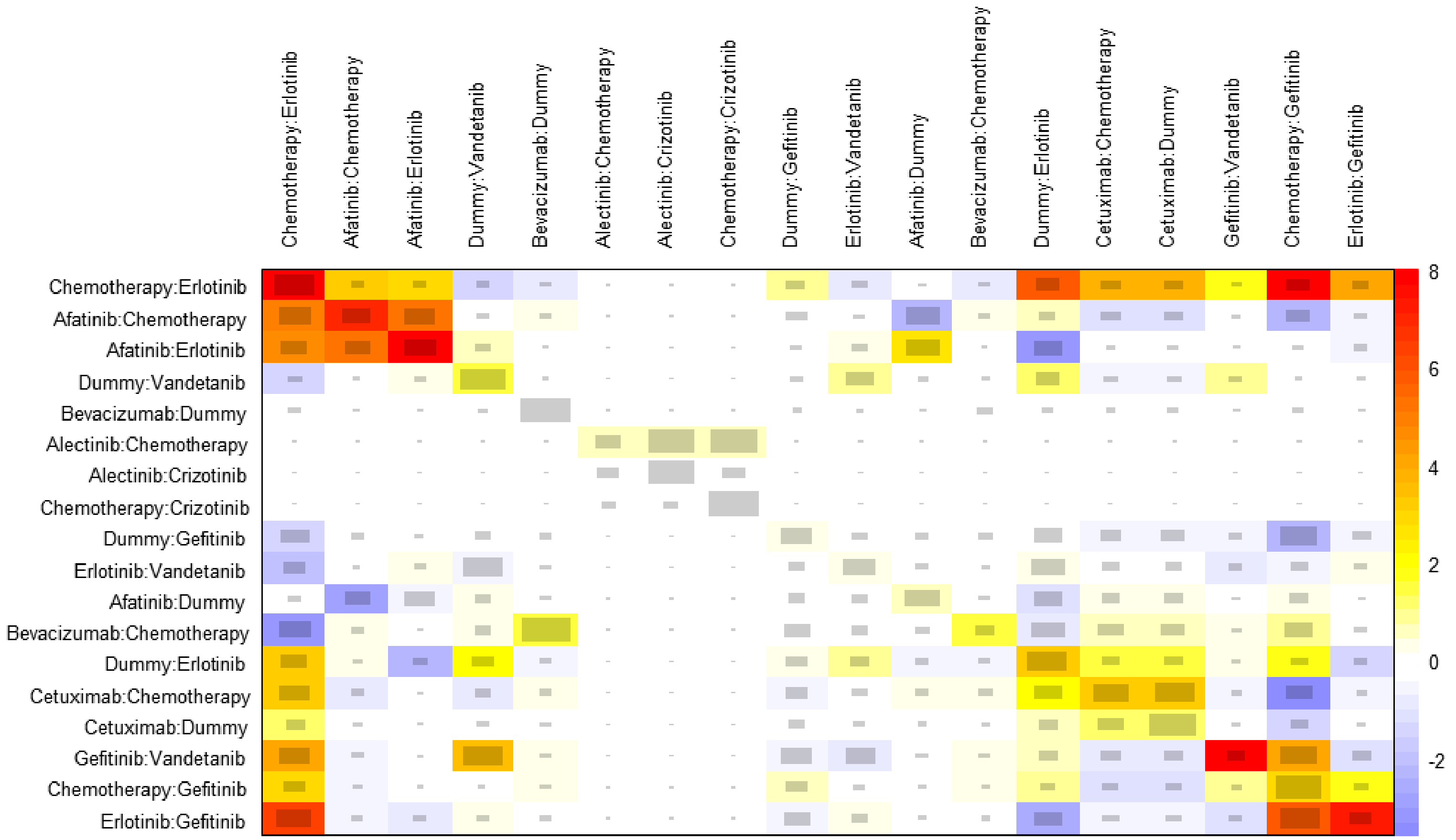

The gray square represents the contribution of the direct estimate of the column-defining comparison to the network estimate in the row. The colors on the diagonal display the inconsistency contribution of the corresponding comparison. The colors on the off-diagonal are related to the change in inconsistency between direct and indirect estimate in a network estimate in the row after relaxing the consistency assumption for the effect of one comparison in the column.

Figure S4. Node-splitting analysis of inconsistency for the comparison of overall response rate in the Bayesian approach

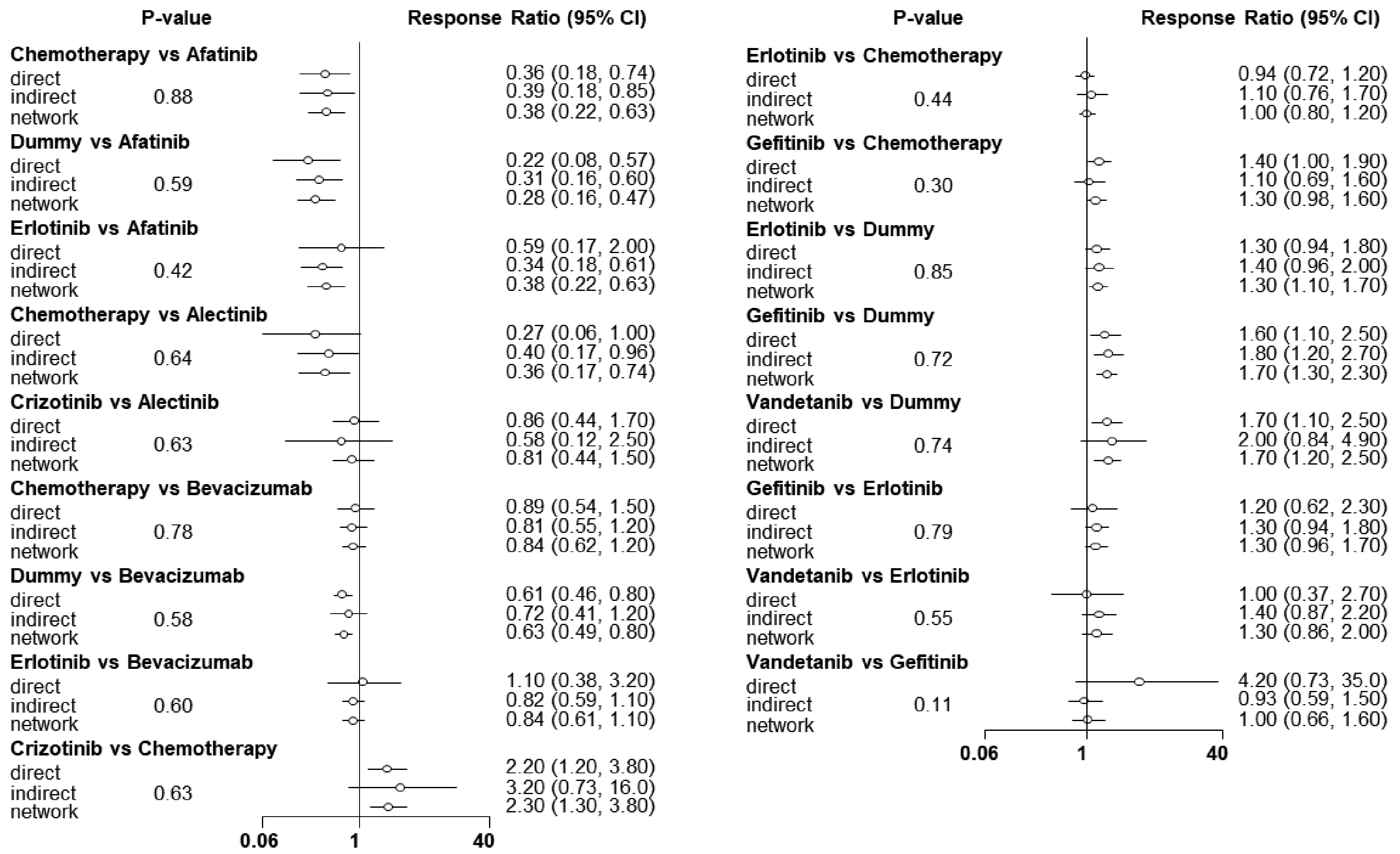

Figure S5. Node-splitting analysis of inconsistency for the comparison of progression-free survival in the Bayesian approach

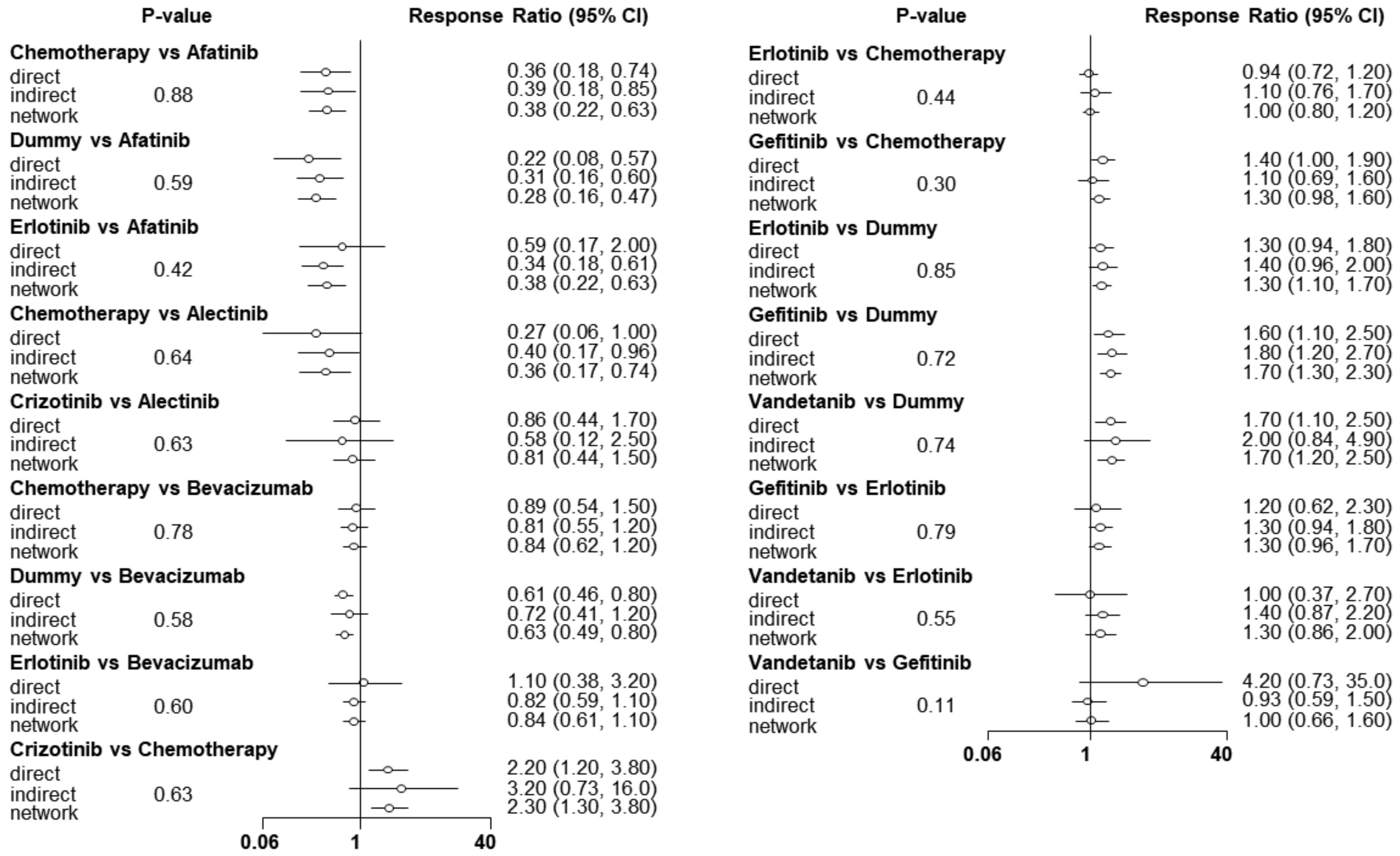

Figure S6. Convergence diagnostics for the comparison of overall response rate

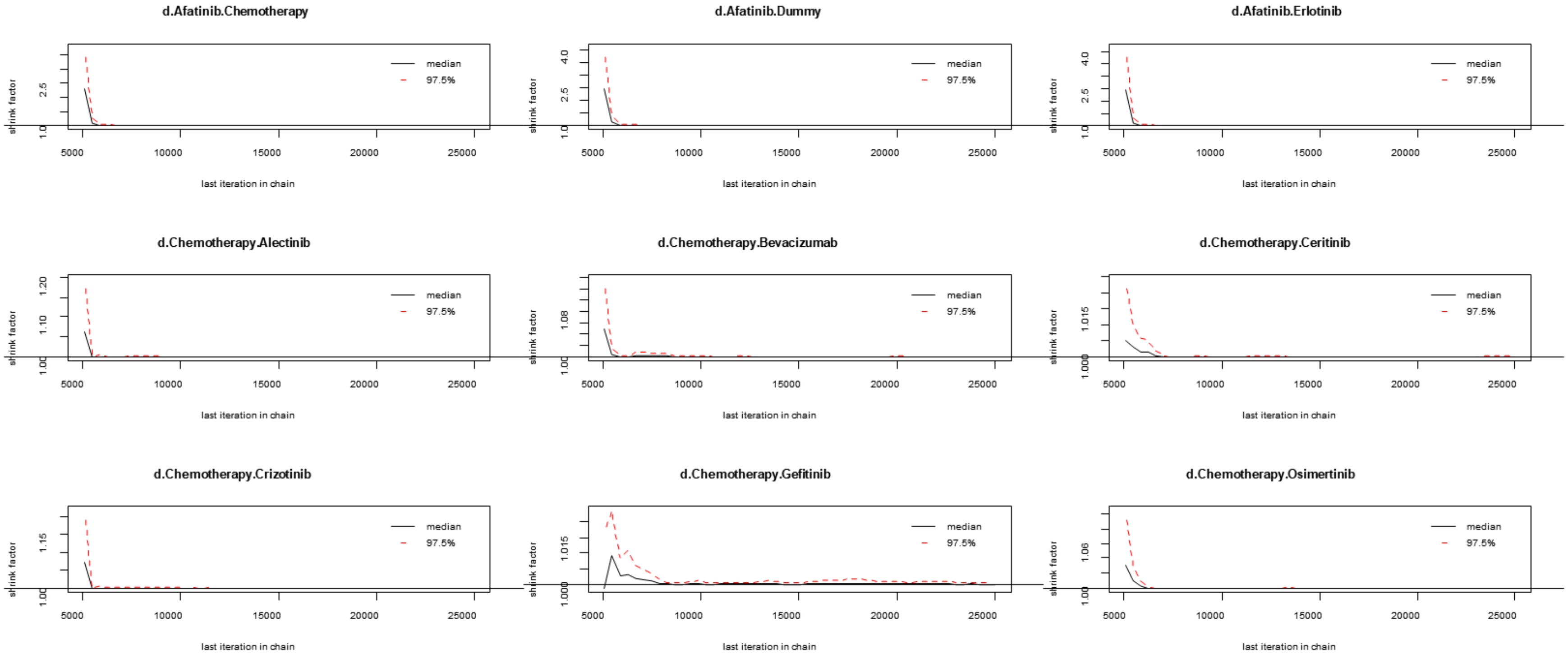

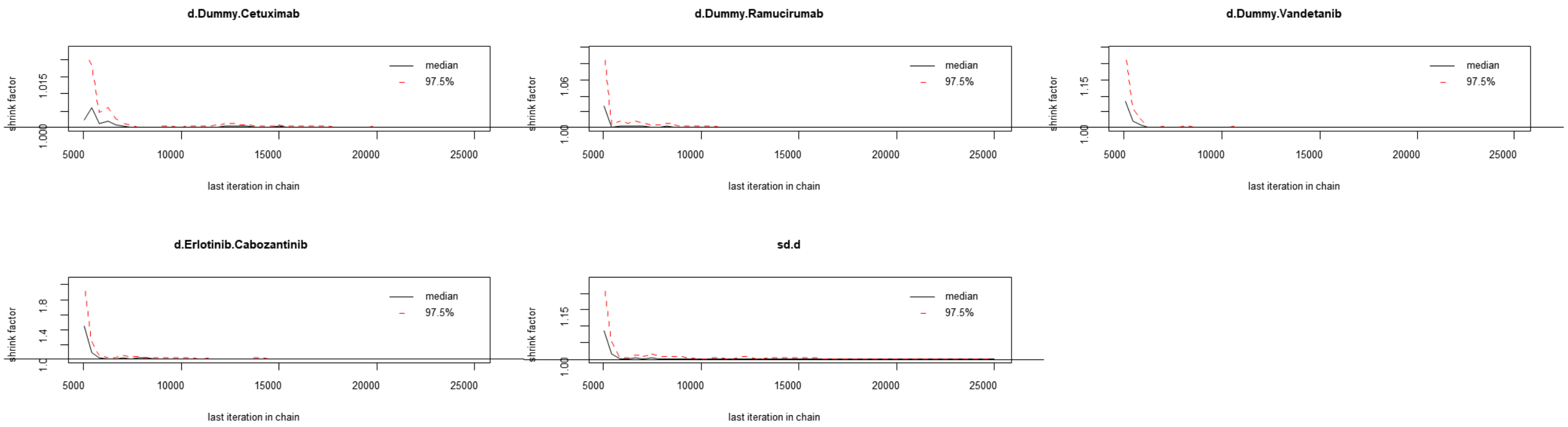

The Gelman diagram gives the scale reduction factors for each parameter. A factor of 1 means that between chain variance and within chain variance are equal, larger values mean that there is still a notable difference between chains.

Figure S7. Convergence diagnostics for the comparison of progression-free survival

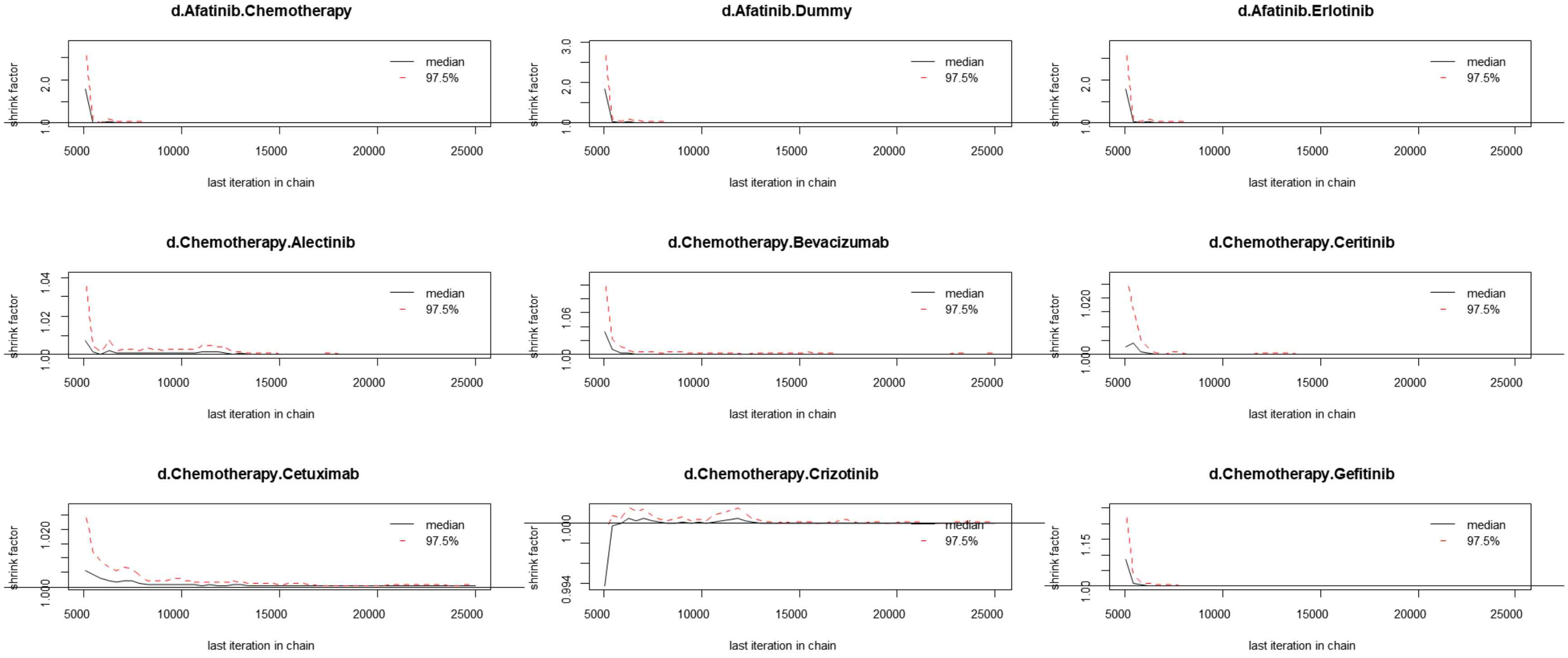

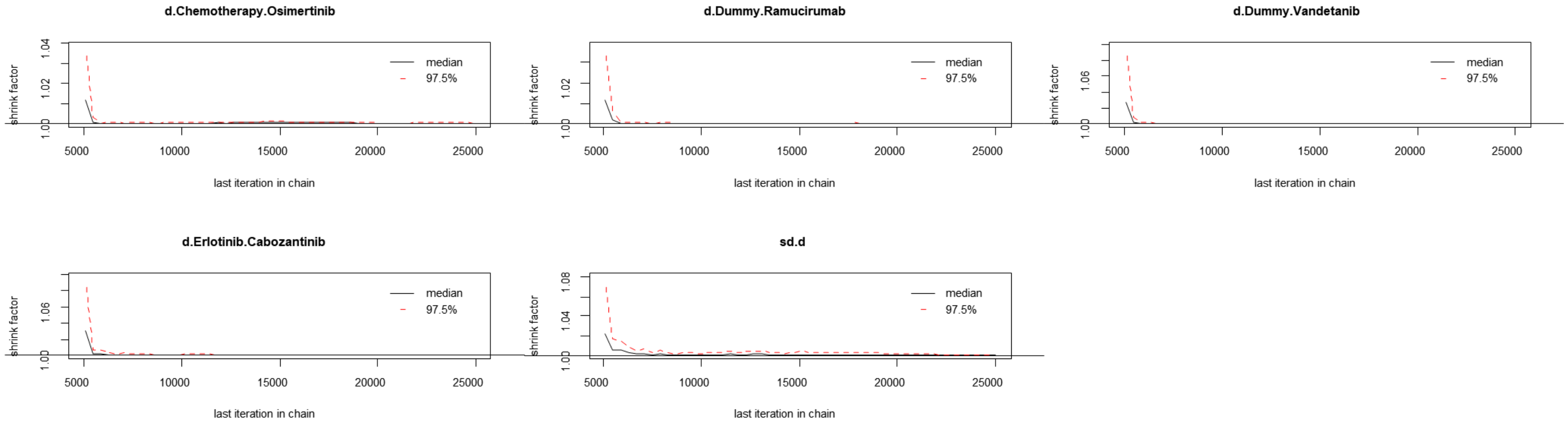

The Gelman diagram gives the scale reduction factors for each parameter. A factor of 1 means that between chain variance and within chain variance are equal, larger values mean that there is still a notable difference between chains.

Figure S8. Treatment ranking plots according to overall response rate and progression-free survival

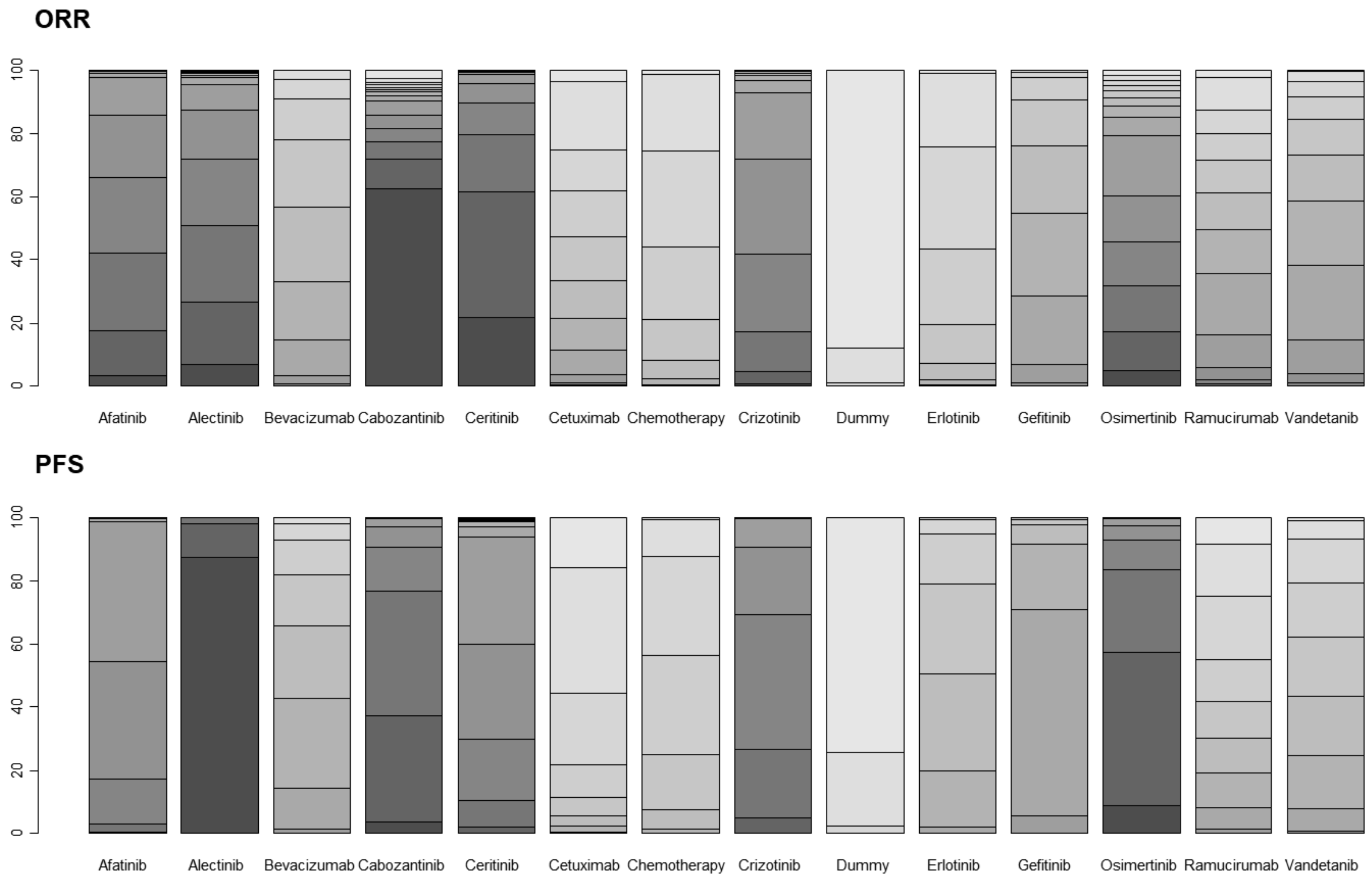

The treatment ranking was based on their probabilities in terms of both overall response rate (ORR) and progression-free survival (PFS) outcomes. In the plot, each vertical bar indicates probabilities that a specific treatment has different ranks. Darker area represents probabilities of having a higher rank. Thus, black area shows the probabilities of having the best treatment.

Figure S9. Cluster ranking plot based on SUCRA values of the overall response rate and progression-free survival of treatments

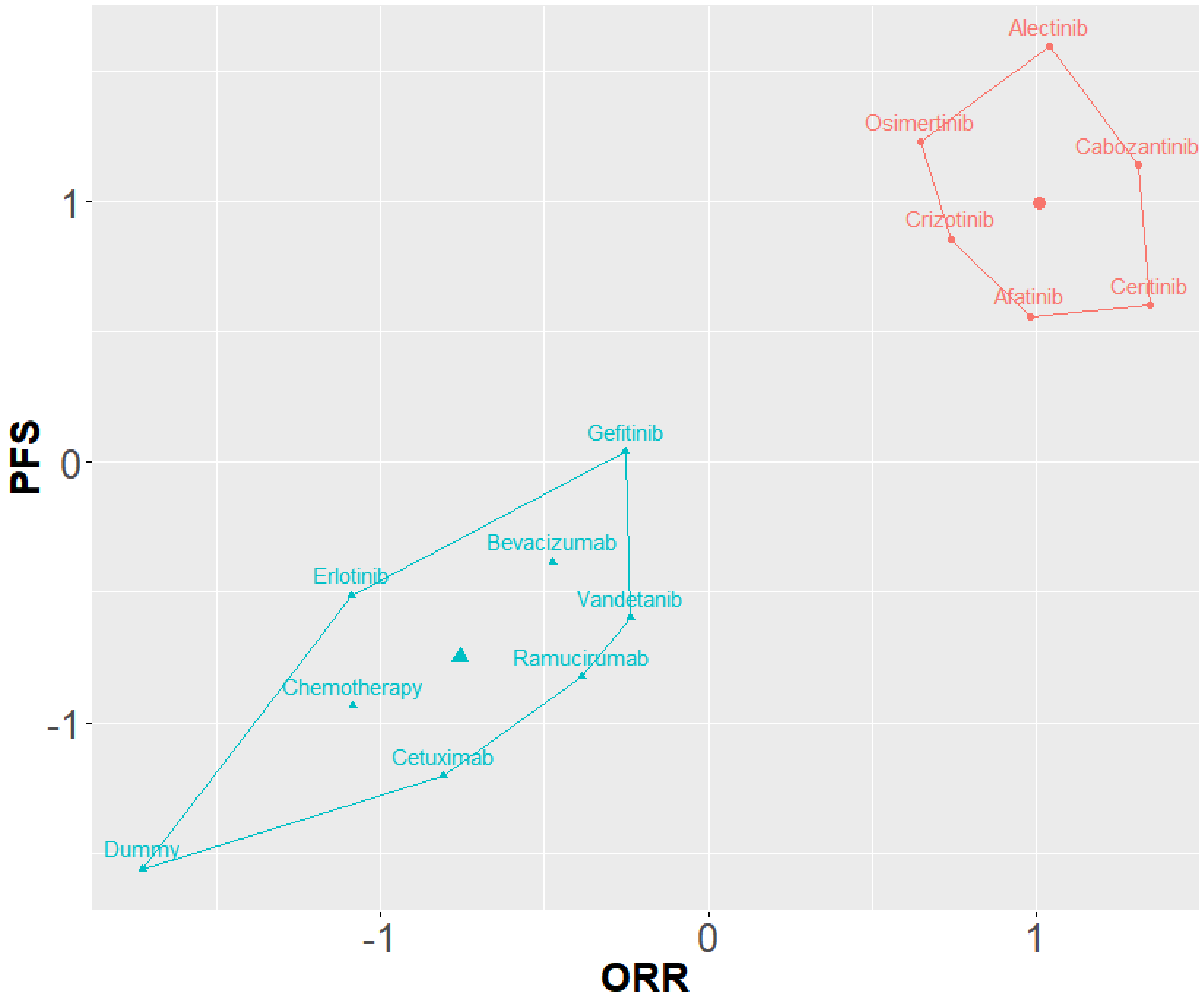

Correlation coefficient = 0.91

Figure S10. Two-dimensional graphs for response ratio and hazard ratio compared to dummy group

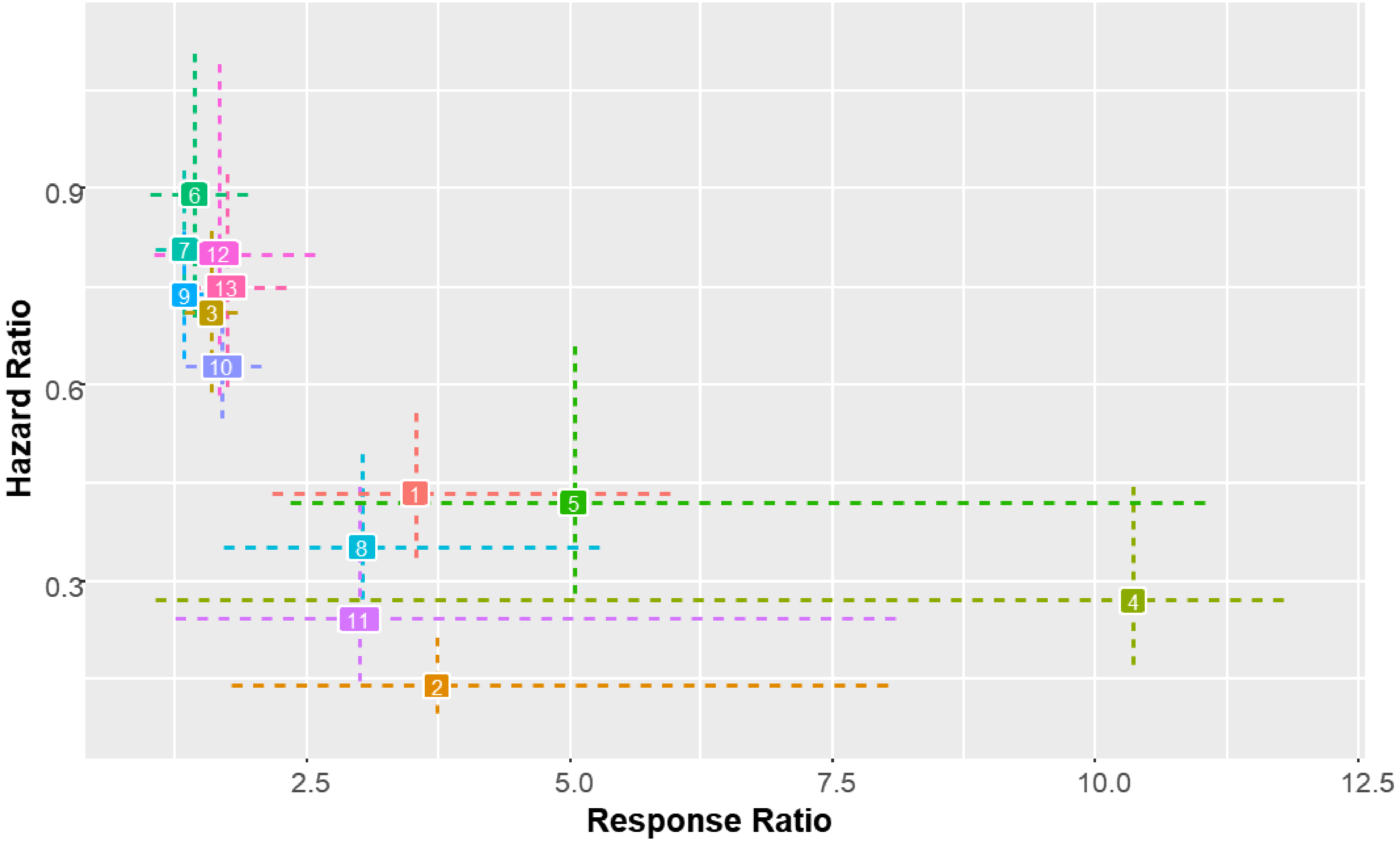

1, Afatinb; 2, Alectinib; 3; Bevacizumab; 4, Cabozantinib; 5, Ceritinib; 6, Cetuximab; 7, Chemotherapy; 8, Crizotinib; 9, Erlotinib; 10, Gefetinib; 11, Osimertinib; 12, Ramucizumab; 13, Vandetanib

Table S1. General characteristics of the studies included in the final analysis

Study	Period, country	Arm 1				Arm 2				HR (95% CI)
		Regimen	No.	ORR (%)	Median PFS (months)	Regimen	No.	ORR (%)	Median PFS (months)	
Johnson 2004	North America	Bevacizumab + Carboplatin + Paclitaxel	35	31.4	7.4	Carboplatin + Paclitaxel	32	18.8	4.2	-
Cufer 2005 (SIGN)	10/2003-06/2004 (12 countries)	Gefitinib	68	-	3	Docetaxel	73	-	3.4	0.94 (0.64-1.39)
Arnold 2007 (BR.20)	05/2003-03/2006 (Canada)	Vandetanib	53	-	2.7	Placebo	54	-	2.8	1.01 (0.64-1.59) [b]
Butts 2007	US, Canada	Gemcitabine + Cisplatin/ Carboplatin + Cetuximab	65	27.7	5.09	Gemcitabine + Cisplatin/ Carboplatin	66	18.2	4.21	-
Gatzemei 2007 (TALENT)	11/2001-09/2002 (27 countries)	Erlotinib + Gemcitabine	580	31.6	-	Placebo + Gemcitabine	579	39.9	-	-
Herbst 2007	10/2004-11/2005	Bevacizumab + Docetaxel/ Pemetrexed	40	12.5	4.8 [a]	Placebo + Docetaxel/ Pemetrexed	41	12.2	3 [a]	-
Heymach 2007	05/2003-07/2004 (3 countries)	Vandetanib + Docetaxel	44	18.2	3.97 [a]	Placebo + Docetaxel	41	12.2	2.8 [a]	0.83 (0.50-1.36)
Crino 2008 (INVITE)	07/2004-12/2005 (10 countries)	Gefitinib	97	3.1	2.7	Vinorelbine	99	5.1	2.9	1.19 (0.85-1.65)
Kelly 2008 (SWOG 0023)	06/2001-04/2005	Placebo	125	72.0	11.7	Gefitinib	118	68.6	8.3	0.80 (0.58-1.10)
Kim 2008 (INTEREST)	02/2004-03/2007 (24 countries)	Gefitinib	723	9.1	2.2	Docetaxel	710	7.6	2.7	1.04 (0.93-1.18)
Lilenbaum 2008	-	Erlotinib	52	3.8	1.91	Carboplatin/ Paclitaxel	51	11.8	3.52	1.45 (0.98-2.15)
Maruyama 2008 (V-15-32)	09/2003-01/2006 (Japan)	Gefitinib	245	22.4	2	Docetaxel	244	12.7	2	0.81 (0.65-1.02)
Rrosell 2008	02/2002-05/2003	Cetuximab + Cisplatin/ Vinorelbine	43	34.9	5	Cisplatin/ Vinorelbine	43	27.9	4.6	0.71 (0.4-1.2)
Goss 2009	09/2004-12/2006 (5 countries)	Gefitinib	100	6.0	1.43	Placebo	101	1.0	1.37	0.82 (0.60-1.12)

Study	Period, country	Arm 1				Arm 2				HR (95% CI)
		Regimen	No.	ORR (%)	Median PFS (months)	Regimen	No.	ORR (%)	Median PFS (months)	
Mok 2009	06/2006-04/1007 (7 countries)	Gemcitabine + Cisplatin/ Carboplatin + Erlotinib	76	35.5	6.86	Gemcitabine + Cisplatin/ Carboplatin + Placebo	78	24.4	5.46	0.47 (0.33-0.68)
Natale 2009	05/2003-08/2004 (6 countries)	Vandetanib	83	8.4	2.6	Gefitinib	85	1.2	1.9	0.69 (0.50-0.96)
Reck 2009 (AVAiL)	02/2005-08/2006 (20 countries)	Bevacizumab + Cisplatin + Gemcitabine	351	30.5	6.5 [a]	Cisplatin + Gemcitabine	347	20.2	6.1 [a]	0.82 (0.68-0.98)
Hong 2010	09/2005-08/2008 (Korea)	Gefitinib	20	26.3	3.5 [a]	Erlotinib	17	11.8	4.4 [a]	-
Lee 2010	09/2005-09/2006 (Korea)	Gefitinib	82	28.0	3.3	Docetaxel	79	7.6	3.4	0.73 (0.50-1.07) [b]
Lynch 2010 (BMS099)	01/2005-11/2006 (US)	Cetuximab + Paclitaxel/ Docetabaxel + Carboplatin	325	25.7	4.4	Paclitaxel/ Docetaxel + Carboplatin	320	16.9	4.24	0.90 (0.76-1.07)
Maemondo 2010	03/2006-05/2009 (Japan)	Gefitinib	114	73.7	10.8	Carboplatin + Paclitaxel	114	30.7	5.4	0.30 (0.22-0.41)
Mitsudomi 2010 (WJTOG 3405)	03/2006-06/2009 (Japan)	Gefitinib	86	61.6	9.2	Cisplatin + Docetaxel	86	32.6	6.3	0.49 (0.34-0.71)
Morere 2010 (IFCT-0301)	12/2004-06/2007	Docetaxel	41	7.3	2 [a]	Gefitinib	43	0.0	1.9 [a]	0.67 (0.43-1.05)
Takeda 2010 (WJTOG 0203)	03/2003-05/2005 (Japan)	Chemotherapy -> Gefitinib	298	34.2	4.6	Chemotherapy -> Chemotherapy	297	29.3	4.3	0.68 (0.57-0.80)
Brahmer 2011 (ECOG 4599)	08/2002-09/2006 (US)	Paclitaxel + Carboplatin + Bevacizumab	191	28.8	6.3	Paclitaxel + Carboplatin	230	15.7	4.3	0.64 (0.53-0.78)
			190	41.1	6.2		162	20.4	5.3	0.71 (0.57-0.88)
De Boer 2011	01/2007-03/2008	Vandetanib + Pemetrexed	256	19.1	4.11	Placebo + Pemetrexed	278	7.9	2.78	0.86 (0.71-1.04)b
GaAfatinibr 2011 (NCT00091156)	05/2004-07/2008 (5 countries)	Gefitinib	86	11.6	4.1	Placebo	87	1.1	2.9	0.61 (0.45-0.83)
Chen 2012	02/2007-07/2008	Erlotinib	57	22.8	4.57	Vinorelbine	56	8.9	2.53	0.64

Study	Period, country	Arm 1				Arm 2				HR (95% CI)
		Regimen	No.	ORR (%)	Median PFS (months)	Regimen	No.	ORR (%)	Median PFS (months)	
	(Taiwan)									(0.43-0.96)
Kelly 2012	01/2008-07/2009 (6 countries)	Pralatrexate	100	2.0	3.4	Erlotinib	101	6.9	2.8	0.91 (0.63-1.32)
Kim 2012	08/2007-10/2008	Gefitinib	48	47.9	4.9	Erlotinib	48	39.6	3.1	-
Lee 2012 (TOPICAL)	04/2005-04/2009 (UK)	Erlotinib	350	4.3	2.8	Placebo	320	2.2	2.6	0.80 (0.68-0.93)
Niho 2012 (JO19907)	04/2007-03/2008	Carboplatin/ Paclitaxel + Bevacizumab	121	60.7	6.9	Carboplatin/ Paclitaxel	59	31.0	5.9	0.61 (0.42-0.89)
Perol 2012	07/2006-06/2009	Chemotherapy -> Erlotinib	155	52.3	2.9 [a]	Chemotherapy	155	51.0	1.9 [a]	0.69 (0.54-0.88)
Rosell 2012 (EURTAC)	02/2007-01/2011	Erlotinib	86	58.1	9.7	Chemotherapy	87	14.9	5.2	0.37 (0.25-0.54)
Spigel 2012	-	Ixabepilone/ Carboplatin	42	28.6	5.3	Ixabepilone/ Carboplatin + Bevacizumab	40	50.0	6.7	-
Sun 2012 (KCSG-LU08-01)	07/2008-06/2010 (Korea)	Gefitinib	68	58.8	9	Pemetrexed	67	22.4	3	0.53 (0.36-0.80)
Boutsikou 2013	-	Erlotinib + Chemotherapy	52	44.2	-	Bevacizumab + Chemotherapy	56	39.3	-	-
Bylicki 2013 (IFCT-GFPC 05-02)	07/2006-06/2009 (France)	Erlotinib	116	-	4.2	Cisplatin + Gemcitabine	130	-	3.9	0.83 (0.64-1.09)
Dai 2013	01/2010-08/2012 (China)	Gefitinib	23	17.4	4.4	Pemetrexed	23	13.0	3.1	-
Fiala 2013	2008-2011 (Czech Republic)	Chemotherapy	150	18.2	-	Erlotinib	129	7.6	-	-
Garassino 2013 (TAILOR)	10/2007-03/2012 (Italy)	Docetaxel	110	13.6	2.9	Erlotinib	112	2.7	2.4	0.71 (0.53-0.95)
Gregorc 2013 (PROSE)	-	Erlotinib	134	-	2.5	Chemotherapy	129	-	4.8	1.26 (0.94-1.69)

Study	Period, country	Arm 1				Arm 2				HR (95% CI)
		Regimen	No.	ORR (%)	Median PFS (months)	Regimen	No.	ORR (%)	Median PFS (months)	
				-	1.7			-	2.8	1.51 (0.96-2.38)
Inoue 2013 (NEJ002)	-	Gefitinib	114	-	10.8	Carboplatin/ Paclitaxel	114	-	5.4	0.33 (0.24-0.44)
Lee 2013	11/2007-07/2010 (8 countries)	Erlotinib	82	29.3	3.8 [a]	Pemetrexed	80	10.0	4.4 [a]	0.99 (0.70-1.40)
Wang 2013	08/2008-09/2011	Pemetrexed + Carboplatin + Gefitinib	30	-	39.8	Pemetrexed + Carboplatin	30	-	27	0.37 (0.16-0.85)
Auliac 2014 (GFPC 10.02)	06/2011-02/2013	Erlotinib + Docetaxel	73	4.1	2.2	Docetaxel	74	1.4	2.5	-
Gridelli 2014	10/2008-12/2011 (Italy)	Vandetanib + Gemcitabine	61	14.8	6.1	Placebo + Gemcitabine	63	12.7	5.63	-
Heigener 2014	06/2006-08/2008 (Germany)	Erlotinib	144	7.6	2.4	Carboplatin + Vinorelbine	140	28.6	4.6	1.6 (1.22-2.09)
Kawaguchi2014 (DELTA)	08/2009-07/2012 (Japan)	Erlotinib	150	17.0	2.2	Docetaxel	151	17.9	3.2	1.22 (0.97-1.55)
Seto 2014 (JO25567)	02/2011-03/2012	Bevacizumab +Erlotinib	77	69.3	16	Erlotinib	77	63.6	9.7	0.54 (0.36-0.79)
Choi 2015	04/2010-12/2011 (Korea)	Gefitinib + Paclitaxel + Carboplatin	44	40.9	4.1	Paclitaxel + Carboplatin	46	39.1	4.1	0.94 (0.61-1.45)
Doebele 2015	10/2010-10/2011	Ramucirumab + Pemetrexed + Carboplatin/ Cisplatin	69	49.3	7.2	Pemetrexed + Carboplatin/ Cisplatin	71	38.0	5.6	0.75 (0.52-1.09) [b]
Halmos 2015	2008-2012	Erlotinib	22	13.6	5.5	Pemetrexed/ Docetaxel	24	16.7	4.4	-
Michael 2015 (GATE)	06/2009-09/2011 (Australia)	Gemcitabine	28	7.1	1.9	Erlotinib + Gemcitabine	26	3.8	2.4	1.3 (0.63-2.68)
Neal 2015 (E1512)	-	Carboplatin	39	-	3.9 [a]	Erlotinib	39	-	1.9 [a]	0.33 (0.18-0.61)bc
Pujol 2015 (IFCT-0802)	09/2009-10/2011 (French)	Bevacizumab	37	91.9	5.3	Chemotherapy	37	83.8	5.5	1.05 (0.67-1.67)
Seto 2015 (WJOG 5910L)	Japan	Bevacizumab + Docetaxel	50	-	4.4	Docetaxel	50	-	3.4	0.71 (0.47-1.09)

Study	Period, country	Arm 1				Arm 2				HR (95% CI)
		Regimen	No.	ORR (%)	Median PFS (months)	Regimen	No.	ORR (%)	Median PFS (months)	
Thomas 2015 (INNOVATIONS)	11/2007-08/2009 (Germany)	Erlotinib + Bevacizumab	111	11.7	3.5	Cisplatin + Gemcitabine + Bevacizumab	113	36.3	6.9	1.85 (1.39-2.45)
Wang 2015	01/2010-08/2013 (China)	Gefitinib	37	54.1	-	Chemotherapy	34	47.1	-	-
Karayama 2016	Japan	Bevacizumab + Pemetrexed	45	-	11.5	Pemetrexed	35	-	7.3	0.73 (0.44-1.19)
Kim 2016 (NCT01783834)	02/2008-06/2014 (Korea)	Pemetrexed	47	17.8	2	Gefitinib	48	8.7	2	-
Lee 2016 (NCT01502202)	06/2012-12/2014 (Korea)	Gefitinib	39	79.5	12.8	Placebo	37	54.1	7	0.51 (0.33-0.81) [b]
Urata 2016	-	Gefitinib	280	46.1	6.5	Erlotinib	279	44.1	7.5	1.13 (0.94-1.4)
Gautschi 2017	-	Bevacizumab + Pemetrexed	77	62.3	6.9	Pemetrexed	52	44.2	5.6	0.7 (0.5-1.0)
Herbst 2017 (NCT00946712)	-	Cetuximab	656	-	4.6	Chemotherapy	657	-	4.5	0.99 (0.88-1.10)
Hida 2017 (J-ALEX)	11/2013-08/2015 (Japan)	Alectinib	103	85.4	NA	Crizotinib	104	70.2	10.2	0.34 (0.17-0.71)
Prabhash 2017	India	Gefitinib	-	-	8.43	Pemetrexed + Cisplatin/ Carboplatin	-	-	5.6	0.66 (0.51-0.85)
Saruwatari 2017	01/2010-03/2016 (Japan)	Erlotinib	13	84.6	10.2	Gefitinib	10	60.0	7.2	-
Spigel 2017	-	Erlotinib + Sorafenib	24	8.3	3.1	Sorafenib	28	3.6	1.9	-
Wakelee 2017 (GO27821)	-	Bevacizumab + Onartuzumab + Paclitaxel	69	49.3	5	Pemetrexed + Onartuzumab + Paclitaxel	59	27.1	4.8	-
		Bevacizumab + Placebo + Paclitaxel	70	42.9	6.8	Pemetrexed + Placebo + Paclitaxel	61	36.1	6.9	-
Wakelee 2017 (NCT00596648)	-	Erlotinib + Cabazantinib	15	6.7	3.9	Cabozantinib	13	0.0	1.9	-
Gupta 2018	11/2014-03/2017	Erlotinib	100	-	4.5	Pemetrexed	100	-	4.46	0.98 (0.71-1.37)

Study	Period, country	Arm 1				Arm 2				HR (95% CI)
		Regimen	No.	ORR (%)	Median PFS (months)	Regimen	No.	ORR (%)	Median PFS (months)	
Spigel 2018 (ToPPS)	06/2009-07/2014 (US)	Bevacizumab + Pemetrexed	63	30.5	4 [a]	Pemetrexed	48	14.6	2.8 [a]	-
NCT00741988	09/2008-09/2010 (US)	Ixabepilone + Carboplatin + Bevacizumab	40	50.0	6.7	Ixabepilone + Carboplatin	42	28.9	5.3	-
NCT00085839	02/2004-03/2007 (US)	Erlotinib	52	3.8	1.91	Paclitaxel + Carboplatin	51	11.8	3.52	1.45 (0.98-2.15)
NCT00892710	06/2009-07/2014 (US)	Pemetrexed + Bevacizumab	63	30.5	4	Pemetrexed	48	14.6	2.8	-
NCT00948675	09/2009-01/2013 (US)	Paclitaxel + Carboplatin + Bevacizumab	179	27.4	5.45	Pemetrexed + Carboplatin	182	23.6	4.44	-
NCT00095199 (Kim 2013)	01/2005-07/2011 (Canada, US)	Cetuximab + Pemetrexed	301	6.6	2.89	Pemetrexed	304	4.3	2.76	1.03 (0.87-1.21)
		Cetuximab + DoCetuximabaxel	167	7.8	2.37	Docetaxel	166	6.6	1.54	0.91 (0.73-1.13)
NCT00112294	12/2004-04/2007 (US)	Carboplatin + Cetuximab + Taxane	338	25.7	4.4	Carboplatin + Taxane	338	17.2	4.24	0.90 (0.76-1.07)
NCT00130728 (Herbst 2011)	06/2005-07/2008 (16 countries)	Bevacizumab + Erlotinib	319	11.9	3.4	Placebo + Erlotinib	317	6.0	1.7	0.62 (0.52-0.75)
NCT00148798 (Pirker 2009)	10/2004-07/2007 (28 countries)	Cetuximab + Chemotherapy	557	36.4	4.8	Chemotherapy	568	29.2	4.8	0.94 (0.83-1.08)
NCT00257608 (Kabbinavar 2014)	01/2006-11/2014 (17 countries)	Erlotinib + Bevacizumab	370	-	4.8	Placebo + Bevacizumab	373	-	3.7	0.71 (0.58-0.86)
NCT00283244 (Stinchcombe 2011)	03/2006-09/2014 (US)	Erlotinib + Gemcitabine	51	21.6	4.1	Gemcitabine	44	6.8	3.7	-
NCT00312377 (Herbst 2010)	05/2006-08/2008 (25 countries)	Vandetanib + Docetaxel	694	17.3	4.04	Placebo + Docetaxel	697	10.2	3.27	0.79 (0.71-0.88) [b]
NCT00322452 (Mok 2009)	03/2006-04/2008 (9 countries)	Gefitinib	609	43.0	5.7	Carboplatin/ Paclitaxel	608	32.2	5.8	0.74 (0.65-0.85)
NCT00364351	08/2006-09/2008	Vandetanib	623	12.0	11.3	Erlotinib	617	12.0	8.9	0.98

Study	Period, country	Arm 1				Arm 2				HR (95% CI)
		Regimen	No.	ORR (%)	Median PFS (months)	Regimen	No.	ORR (%)	Median PFS (months)	
	(22 countries)									(0.87-1.10)
NCT00373425 (Kelly 2015)	09/2006-04/2013 (19 countries)	Erlotinib	623	-	55	Placebo	350	-	56.2	0.94 (0.78-1.14)
NCT00404924 (ZD6474)	11/2006-10/2009 (22 countries)	Vandetanib + BSC	617	2.6	1.9	Placebo + BSC	307	0.7	1.8	-
NCT00409006	02/2007-08/2009 (China, Korea, Taiwan)	Gefitinib + Pemetrexed + Cisplatin	39	46.2	9.95	Pemetrexed + Cisplatin	31	35.5	6.83	0.53 (0.27-1.04)
NCT00418886 (ZEAL)	01/2007-09/2008 (21 countries)	Vandetanib + Pemetrexed	256	19.1	17.6	Placebo + Pemetrexed	278	7.9	11.9	0.86 (0.69-1.06)
NCT00447057 (Dittrich 2014)	03/2007-07/2010 (5 countries)	Erlotinib + Pemetrexed	60	17.1	3.19	Pemetrexed	70	10.8	2.89	0.63 (0.44-0.90)
NCT00518011	08/2007-02/2009 (Australia)	Gemcitabine	8	0.0	5.44	Erlotinib + Gemcitabine	7	5.7	5.69	1.84 (0.48-7.03)
NCT00531960 (Ciuleanu 2013)	01/2008-01/2010 (13 countries)	Erlotinib + Bevacizumab	63	27.0	5.5	Erlotinib + Chemotherapy	61	44.3	8.1	1.05 (0.70-1.59)
NCT00550173	11/2007-01/2012 (7 countries)	Erlotinib + Pemetrexed	76	44.7	7.4	Pemetrexed	80	10.0	4.4	0.58 (0.39-0.85)
NCT00556322 (Ciuleanu 2012)	03/2006-06/2012 (27 countries)	Erlotinib	203	7.9	6.3	Chemotherapy	221	6.3	8.6	1.19 (0.97-1.46)
NCT00556712 (Cappuzzo 2010)	01/2006-11/2010 (28 countries)	Erlotinib	437	11.9	12.3	Placebo	447	5.4	11.1	0.71 (0.62-0.82)
NCT00606502	01/2008-06/2009 (6 countries)	Erlotinib	101	7.1	2.8	Pralatrexate	100	2.1	3.4	-
NCT00609804	03/2008-05/2012 (US)	Erlotinib + Sorafenib	24	8.3	3.1	Sorafenib	28	3.6	1.9	-
NCT00656136 (Miller 2012)	04/2008-10/2013 (15 countries)	Afatinib + Best support care	390	7.4	3.29	Placebo + Best support care	195	0.5	1.08	0.38 (0.31-0.48)
NCT00660816	01/2008-04/2013 (US)	Erlotinib + Pemetrexed/ Docetaxel	22	16.7	4.4	Pemetrexed/ Docetaxel	24	13.3	5.5	-
NCT00687297 (Aisner 2013)	04/2008-01/2011	Placebo + Docetaxel + Carboplatin	82	18.3	4.2	Vandetanib + Docetaxel + Carboplatin	80	18.8	4.5	1.49 (1.07-2.07)

Study	Period, country	Arm 1				Arm 2				HR (95% CI)
		Regimen	No.	ORR (%)	Median PFS (months)	Regimen	No.	ORR (%)	Median PFS (months)	
NCT00753714 (ZELIG)	10/2008-04/2011 (Italy)	Vandetanib + Gemcitabine	61	14.8	6.1	Placebo + Gemcitabine	63	12.7	5.6	-
NCT00770588	09/2008-08/2009	Gefitinib	148	23.6	4.8	Placebo	148	0.7	2.6	0.42 (0.33-0.55)
NCT00777179	10/2008-01/2010 (Korea)	Vandetanib	75	18.7	2.7	Placebo	42	2.4	1.7	-
NCT00800202 (BRAIN)	04/2009-10/2012 (France)	Erlotinib + Bevacizumab	22	12.5	6.3	Bevacizumab + Paclitaxel + Carboplatin	60	62.7	6.7	-
NCT00883779	04/2009-12/2014 (7 countries)	Erlotinib	226	17.7	7.6	Placebo	225	43.1	6	0.57 (0.46-0.70)
NCT00932893 (Shaw 2016)	09/2009-03/2012 (22 countries)	Crizotinib	173	65.3	7.7	Pemetrexed/ Docetaxel	174	19.5	3	0.49 (0.37-0.64)
NCT00949650 (LUX-lung 6)	08/2009-02/2012 (25 countries)	Afatinib	230	56.5	11.17	Pemetrexed/ Cisplatin	115	22.6	6.9	0.58 (0.43-0.78)
NCT01085136 (LUX-Lung 5)	02/2010-10/2013 (24 countries)	Afatinib + Paclitaxel	134	32.1	5.55	Chemotherapy	68	13.2	2.89	0.61 (0.44-0.85)
NCT01121393 (LUX-lung 3)	04/2010-12/2013 (3 countries)	Afatinib	242	67.8	11.01	Gemcitabine/ Cisplatin	122	23.0	5.59	0.28 (0.20-0.39)
NCT01154140 (PROFILE 1014)	01/2011-11/2013 (31 countries)	Crizotinib	172	74.4	10.9	Chemotherapy	171	45.0	7	0.45 (0.35-0.60)
NCT01160744 (Thomas 2017)	09/2010-01/2014 (6 countries)	Ramucirumab + Gemcitabine + Carboplatin/ Cisplatin	71	46.5	5.6	Gemcitabine + Carboplatin/ Cisplatin	69	24.6	5.4	0.88 (0.60-1.29) [b]
NCT01168973 (Reck 2018)	12/2010-12/2013 (27 countries)	Ramucirumab + Docetaxel	628	29.9	4.5	Placebo + Docetaxel	625	13.6	3	0.76 (0.68-0.86)
NCT01196078	02/2007-12/2010 (Taiwan)	Erlotinib	57	22.8	6.66	Vinorelbine	56	8.9	3.87	-
NCT01342965 (ENSURE)	03/2011-07/2012 (China)	Erlotinib	110	68.2	11	Gemcitabine+Cisplatin	107	39.3	5.5	0.34 (0.22-0.51)
NCT01351415	06/2011-06/2016 (18 countries)	Bevacizumab + Erlotinib/ Docetaxel/ Pemetrexed	245	8.6	5.45	Erlotinib/ Docetaxel/ Pemetrexed	240	6.3	3.98	0.83 (0.68-1.02) [b]

Study	Period, country	Arm 1				Arm 2				HR (95% CI)
		Regimen	No.	ORR (%)	Median PFS (months)	Regimen	No.	ORR (%)	Median PFS (months)	
NCT01523587 (Felip 2017)	03/2012-08/2013 (23 countries)	Afatinib	335	4.8	2.43	Erlotinib	334	3.0	1.94	0.82 (0.68-1.00)
NCT01544179 (Soria 2015)	03/2012-05/2014 (12 countries)	Gefitinib	133	31.6	5.4	Placebo	132	34.1	5.4	0.86 (0.65-1.13)
NCT01565538 (Li 2014)	12/2008-05/2012 (China)	Erlotinib	61	19.7	4.1	Pemetrexed	62	8.1	3.9	0.92 (0.62-1.37)
NCT01639001 (Lu 2016)	09/2012-06/2015 (5 countries)	Crizotinib	104	87.5	11.1	Chemotherapy	103	45.6	6.8	0.40 (0.28-0.57)
NCT01703091 (Hosomi 2015)	12/2012-12/2014 (Japan)	Ramucirumab + Docetaxel	76	28.9	5.2	Placebo + Docetaxel	81	18.5	4.2	0.83 (0.59-1.16)
NCT01708954 (Neal 2016)	02/2013-08/2015 (US)	Cabozantinib	38	0.11	4.3	Erlotinib	38	0.03	1.8	0.39 (0.27-0.55)
NCT01828099 (Soria 2017)	07/2013-06/2016 (30 countries)	Ceritinib	189	72.5	16.6	Chemotherapy	187	26.7	8.1	0.55 (0.42-0.73)
NCT01828112 (Shaw2017)	06/2013-01/2016 (20 countries)	Ceritinib	115	39.1	5.4	Pemetrexed/Docetaxel	116	6.9	1.6	0.49 (0.36-0.67)
NCT01998919	08/2006-11/2011 (8 countries)	Erlotinib + Chemotherapy	76	36.8	30.4	Placebo + Chemotherapy	78	24.4	23.4	0.47 (0.33-0.68)
NCT02075840 (Peters 2017)	08/2014-01/2016 (29 countries)	Alectinib	152	82.9	-	Crizotinib	151	75.5	11.1	0.47 (0.34-0.65)
NCT02151981 (Mok 2017)	08/2014-04/2016 (18 countries)	Osimertinib	279	70.6	10.1	Chemotherapy	140	31.4	4.4	0.30 (0.23-0.41)
NCT02604342	11/2015-01/2017 (13 countries)	Alectinib	72	37.5	9.6	Pemetrexed/Docetaxel	35	11.4	1.4	0.15 (0.08-0.29)

ORR, overall response rate; PFS, progression-free survival; HR, hazard ratio; 95% CI, 95% confidence interval

[a] Recalculated median PFS as the formula: $\text{PFS (months)} = \text{PFS (weeks)} * \frac{7}{30}$

[b] Recalculated 95% CI from different levels of significant α as the formula: $(1-\alpha)\%\ \text{CI for HR} = \exp[\hat{\beta}]: \exp[\hat{\beta} \pm z_{1-\alpha/2} * \sqrt{\widehat{\text{Var}}(\hat{\beta})}$

Table S2. Test for heterogeneity

RR for ORR				HR for PFS			
**Treatment 1**	**Treatment 2**	**Pair-wise I^2 (%)**	**Consi-stency I^2 (%)**	**Treatment 1**	**Treatment 2**	**Pair-wise I^2 (%)**	**Consi-stency I^2 (%)**
Afatinib	Chemotherapy	0.00	0.00	Afatinib	Chemotherapy	90.18	87.65
Afatinib	Dummy	76.89	40.37	Afatinib	Dummy	85.15	66.23
Afatinib	Erlotinib	-	23.85	Afatinib	Erlotinib	-	91.74
Alectinib	Chemotherapy	-	0.00	Alectinib	Chemotherapy	-	0.00
Alectinib	Crizotinib	14.89	43.56	Alectinib	Crizotinib	0.00	0.00
Bevacizumab	Chemotherapy	68.89	61.26	Bevacizumab	Chemotherapy	0.00	0.00
Bevacizumab	Dummy	35.97	26.79	Bevacizumab	Dummy	14.37	11.74
Bevacizumab	Erlotinib	-	26.45	Cabozantinib	Erlotinib	0.00	0.00
Cabozantinib	Erlotinib	-	-	Ceritinib	Chemotherapy	0.00	0.00
Ceritinib	Chemotherapy	86.89	86.79	Cetuximab	Chemotherapy	-	70.69
Cetuximab	Dummy	0.00	0.00	Cetuximab	Dummy	0.00	0.00
Chemotherapy	Crizotinib	87.62	83.92	Chemotherapy	Crizotinib	0.00	0.00
Chemotherapy	Erlotinib	85.10	83.81	Chemotherapy	Erlotinib	85.27	87.00
Chemotherapy	Gefitinib	69.48	72.18	Chemotherapy	Gefitinib	90.87	89.56
Chemotherapy	Osimertinib	-	-	Chemotherapy	Osimertinib	-	-
Dummy	Erlotinib	86.67	86.26	Dummy	Erlotinib	68.06	63.54
Dummy	Gefitinib	90.03	89.89	Dummy	Gefitinib	80.03	78.90
Dummy	Ramucirumab	60.26	60.08	Dummy	Ramucirumab	0.00	0.00
Dummy	Vandetanib	31.91	23.34	Dummy	Vandetanib	0.00	29.46
Erlotinib	Gefitinib	0.00	37.39	Erlotinib	Gefitinib	-	89.04
Erlotinib	Vandetanib	-	68.08	Erlotinib	Vandetanib	-	0.00
Gefitinib	Vandetanib	-	65.41	Gefitinib	Vandetanib	-	91.53
Global I^2		78.18	77.64	Global I^2		78.33	78.72

Table S3. Direct pairwise comparative efficacy in the frequentist approach

Left side: **HR for PFS (95% CI)**; right side: **RR for ORR (95% CI)**

Afat						*2.72 (1.59-4.66)*		*3.28 (1.39-7.75)*	1.60 (0.57-4.47)				
	Alec					*3.28 (1.01-10.7)*	1.16 (0.71-1.88)						
		Beva				1.10 (0.74-1.63)		*1.59 (1.27-1.98)*	0.89 (0.39-2.00)				
			Cabo						4.00 (0.42-37.9)				
				Ceri		*3.52 (1.97-6.30)*							
					Cetu			*1.39 (1.02-1.91)*					
0.41 (0.27-0.61)	*0.15 (0.07-0.33)*	1.05 (0.67-1.65)		*0.52 (0.35-0.77)*	0.99 (0.60-1.62)	Chem	*0.46 (0.31-0.70)*		0.96 (0.76-1.21)	*0.66 (0.51-0.84)*	*0.45 (0.22-0.92)*		
	0.42 (0.26-0.68)					*2.23 (1.61-3.09)*	Criz						
0.47 (0.32-0.69)		*0.69 (0.58-0.83)*			0.91 (0.73-1.14)			Dum	*0.72 (0.55-0.96)*	0.75 (0.54-1.05)		*0.58 (0.39-0.86)*	*0.56 (0.40-0.79)*
0.82 (0.49-1.37)			*0.37 (0.23-0.59)*			0.98 (0.85-1.12)		*1.52 (1.28-1.82)*	Erlo	0.84 (0.51-1.40)			1.00 (0.47-2.09)
						1.44 (1.23-1.68)		*1.46 (1.18-1.79)*	0.88 (0.53-1.48)	Gefi			0.14 (0.02-1.24)
						3.33 (1.93-5.77)					Osim		
								1.25 (0.94-1.66)				Ramu	
								1.21 (0.96-1.53)	1.02 (0.62-1.67)	1.45 (0.81-2.58)			Vand

Drugs are reported in alphabetical order. Data are RRs and HRs (95%CI) in the row-defining treatment compared with the column-defining treatment. For ORR, RRs higher than 1 are favour the row-defining treatment. For PFS, HRs lower than 1 are favour the row-defining treatment (the first drug in alphabetical order). Significant results are in italic and underscored. RR, response ratio; HR, hazard ratio; CI, confidence interval; ORR: overall response rate; PFS: progression-free survival. Afat, afatinib; Alec, alectinib; Beva, bevacizumab; Cabo, cabozantinib; Ceri, ceritinib; Cetu, cetuximab; Chem, chemotherapy; Criz, crizotinib; Dum, dummy; Erlo, erlotinib; Gefi, gefitinib; Osim, osimertinib; Ramu, ramucirumab; Vand, vandetanib.

Table S4. Comparative efficacy of targeted therapies for overall response rate in the network meta-analysis based on the frequentist approach

Afat	0.96 0.47-1.94)	*2.03 1.27-3.24)*	0.56 0.06-5.57)	0.72 0.35-1.49)	*2.18 1.26-3.77)*	*2.55 1.67-3.89)*	1.15 0.64-2.05)	*3.05 1.95-4.76)*	*2.25 1.45-3.50)*	*1.90 1.20-2.99)*	1.13 0.49-2.62)	1.76 0.97-3.21)	*1.72 1.01-2.94)*
1.04 0.52-2.11)	Alec	*2.12 1.14-3.93)*	0.59 0.06-6.02)	0.76 0.34-1.70)	*2.28 1.15-4.52)*	*2.66 1.51-4.67)*	1.20 0.76-1.89)	*3.18 1.73-5.83)*	*2.35 1.30-4.26)*	*1.98 1.09-3.61)*	1.18 0.47-2.96)	1.84 0.89-3.80)	1.80 0.92-3.52)
0.49 0.31-0.79)	*0.47 0.25-0.88)*	Beva	0.28 0.03-2.67)	*0.36 0.19-0.67)*	1.08 0.74-1.56)	1.25 0.98-1.61)	*0.57 0.35-0.90)*	*1.50 1.23-1.83)*	1.11 0.86-1.43)	0.93 0.71-1.23)	0.56 0.26-1.20)	0.87 0.56-1.35)	0.85 0.59-1.22)
1.78 0.18-17.6)	1.7 0.17-17.42)	3.60 0.37-34.64)	Cabo	1.28 0.12-13.21)	3.88 0.4-37.93)	4.52 0.47-43.2)	2.04 0.21-20.2)	5.41 0.56-51.7)	4.00 0.42-37.9)	3.37 0.35-32.3)	2.01 0.19-21.5)	3.13 0.32-31.0)	3.06 0.31-29.8)
1.38 0.67-2.84)	1.32 0.59-2.98)	*2.81 1.49-5.29)*	0.78 0.08-8.01)	Ceri	*3.02 1.50-6.07)*	*3.52 1.97-6.30)*	1.59 0.78-3.21)	*4.21 2.26-7.85)*	*3.11 1.69-5.74)*	*2.62 1.41-4.86)*	1.57 0.62-3.97)	*2.44 1.16-5.11)*	*2.38 1.20-4.73)*
0.46 0.27-0.79)	*0.44 0.22-0.87)*	0.93 0.64-1.35)	0.26 0.03-2.52)	*0.33 0.16-0.67)*	Cetu	1.17 0.79-1.72)	*0.53 0.30-0.91)*	*1.39 1.02-1.91)*	1.03 0.71-1.50)	0.87 0.59-1.29)	0.52 0.23-1.18)	0.81 0.49-1.34)	0.79 0.51-1.22)
0.39 0.26-0.60)	*0.38 0.21-0.66)*	0.80 0.62-1.02)	0.22 0.02-2.11)	*0.28 0.16-0.51)*	0.86 0.58-1.26)	Chem	*0.45 0.30-0.67)*	1.20 0.96-1.50)	0.89 0.73-1.07)	*0.74 0.61-0.91)*	*0.45 0.22-0.92)*	0.69 0.44-1.09)	*0.68 0.47-0.98)*
0.87 0.49-1.56)	0.83 0.53-1.32)	*1.77 1.11-2.83)*	0.49 0.05-4.85)	0.63 0.31-1.27)	*1.90 1.09-3.31)*	*2.22 1.49-3.30)*	Criz	*2.65 1.68-4.18)*	*1.96 1.27-3.04)*	*1.65 1.06-2.58)*	0.99 0.43-2.25)	1.54 0.84-2.81)	1.50 0.88-2.57)
0.33 0.21-0.51)	*0.31 0.17-0.58)*	*0.67 0.55-0.81)*	0.19 0.02-1.77)	*0.24 0.13-0.44)*	*0.72 0.52-0.98)*	0.84 0.67-1.05)	*0.38 0.24-0.59)*	Dum	*0.74 0.60-0.91)*	*0.62 0.49-0.79)*	*0.37 0.17-0.79)*	*0.58 0.39-0.86)*	*0.57 0.42-0.77)*
0.44 0.29-0.69)	*0.43 0.23-0.77)*	0.90 0.7-1.16)	0.25 0.03-2.37)	*0.32 0.17-0.59)*	0.97 0.67-1.41)	1.13 0.94-1.36)	*0.51 0.33-0.79)*	*1.35 1.10-1.66)*	Erlo	0.84 0.67-1.06)	0.50 0.24-1.06)	0.78 0.50-1.22)	0.76 0.54-1.08)
0.53 0.33-0.83)	*0.51 0.28-0.92)*	1.07 0.81-1.41)	0.30 0.03-2.85)	*0.38 0.21-0.71)*	1.15 0.78-1.71)	*1.34 1.09-1.65)*	*0.61 0.39-0.95)*	*1.61 1.27-2.03)*	1.19 0.94-1.50)	Gefi	0.60 0.28-1.27)	0.93 0.59-1.48)	0.91 0.63-1.32)
0.88 0.38-2.04)	0.85 0.34-2.12)	1.79 0.83-3.86)	0.50 0.05-5.32)	0.64 0.25-1.62)	1.93 0.85-4.38)	*2.25 1.09-4.64)*	1.01 0.44-2.31)	*2.69 1.26-5.74)*	1.99 0.94-4.20)	1.67 0.79-3.55)	Osim	1.56 0.66-3.66)	1.52 0.68-3.42)
0.57 0.31-1.03)	0.54 0.26-1.12)	1.15 0.74-1.79)	0.32 0.03-3.17)	*0.41 0.20-0.86)*	1.24 0.75-2.06)	1.44 0.91-2.28)	0.65 0.36-1.19)	*1.73 1.16-2.57)*	1.28 0.82-2.00)	1.08 0.68-1.71)	0.64 0.27-1.51)	Ramu	0.98 0.59-1.62)
0.58 0.34-0.99)	0.56 0.28-1.09)	1.18 0.82-1.69)	0.33 0.03-3.18)	*0.42 0.21-0.83)*	1.27 0.82-1.97)	*1.48 1.03-2.13)*	0.67 0.39-1.14)	*1.77 1.30-2.40)*	1.31 0.92-1.85)	1.10 0.76-1.60)	0.66 0.29-1.48)	1.02 0.62-1.69)	Vand

Drugs are reported in alphabetical order. Data in the right-upper triangle are RRs (95%CI) in the row-defining treatment compared with the column-defining treatment. RRs higher than 1 favor the row-defining treatment (the first drug in alphabetical order). RRs for the opposite comparison of ORR are in the left-lower triangle. Each comparison is shown twice in the table, once with drug A versus drug B and once with drug B versus drug A. Significant results are in italic and underscored. RR, response ratio; CI, confidence interval; ORR: overall response rate; Afat, afatinib; Alec, alectinib; Beva, bevacizumab; Cabo, cabozantinib; Ceri, ceritinib; Cetu, cetuximab; Chem, chemotherapy; Criz, crizotinib; Dum, dummy; Erlo, erlotinib; Gefi, gefitinib; Osim, osimertinib; Ramu, ramucirumab; Vand, vandetanib.

Table S5. Comparative efficacy of targeted therapies for progression-free survival in the network meta-analysis based on the frequentist approach

Afat	*3.10<br>1.82-5.27)*	*0.62<br>0.46-0.83)*	1.61<br>0.94-2.78)	1.04<br>0.65-1.67)	*0.49<br>0.35-0.68)*	*0.54<br>0.42-0.70)*	1.24<br>0.83-1.85)	*0.44<br>0.34-0.57)*	*0.59<br>0.46-0.77)*	*0.69<br>0.53-0.91)*	1.80<br>0.98-3.30)	*0.55<br>0.37-0.80)*	*0.58<br>0.42-0.80)*
0.32<br>0.19-0.55)	Alec	*0.20<br>0.12-0.33)*	0.52<br>0.27-1.03)	*0.34<br>0.18-0.62)*	*0.16<br>0.09-0.27)*	*0.17<br>0.11-0.28)*	0.40<br>0.26-0.61)	*0.14<br>0.09-0.23)*	*0.19<br>0.12-0.31)*	*0.22<br>0.14-0.36)*	0.58<br>0.28-1.20)	*0.18<br>0.10-0.31)*	*0.19<br>0.11-0.32)*
1.62<br>1.20-2.20)	*5.02<br>3.01-8.37)*	Beva	*2.62<br>1.56-4.40)*	*1.69<br>1.08-2.64)*	0.79<br>0.61-1.03)	0.88<br>0.71-1.08)	*2.01<br>1.39-2.92)*	*0.71<br>0.60-0.84)*	0.96<br>0.78-1.18)	1.13<br>0.91-1.40)	*2.93<br>1.63-5.26)*	0.89<br>0.64-1.24)	0.95<br>0.73-1.22)
0.62<br>0.36-1.06)	1.92<br>0.98-3.77)	*0.38<br>0.23-0.64)*	Cabo	0.64<br>0.34-1.21)	*0.30<br>0.18-0.52)*	*0.34<br>0.21-0.55)*	0.77<br>0.43-1.37)	*0.27<br>0.17-0.44)*	*0.37<br>0.23-0.59)*	*0.43<br>0.26-0.71)*	1.12<br>0.54-2.33)	*0.34<br>0.19-0.60)*	*0.36<br>0.21-0.61)*
0.96<br>0.60-1.54)	*2.98<br>1.62-5.49)*	*0.59<br>0.38-0.93)*	1.55<br>0.83-2.91)	Ceri	*0.47<br>0.30-0.74)*	*0.52<br>0.35-0.77)*	1.19<br>0.72-1.97)	*0.42<br>0.28-0.64)*	*0.57<br>0.38-0.86)*	0.67<br>0.44-1.01)	1.73<br>0.88-3.41)	*0.53<br>0.32-0.88)*	*0.56<br>0.35-0.89)*
2.04<br>1.48-2.82)	*6.33<br>3.76-10.7)*	1.26<br>0.97-1.63)	*3.30<br>1.94-5.60)*	*2.13<br>1.34-3.36)*	Cetu	1.11<br>0.88-1.40)	*2.54<br>1.72-3.73)*	0.89<br>0.73-1.09)	1.21<br>0.96-1.52)	*1.42<br>1.11-1.81)*	*3.69<br>2.03-6.69)*	1.12<br>0.79-1.59)	1.19<br>0.90-1.58)
1.85<br>1.43-2.39)	*5.72<br>3.59-9.12)*	1.14<br>0.93-1.40)	*2.98<br>1.83-4.87)*	*1.92<br>1.29-2.86)*	0.90<br>0.72-1.14)	Chem	*2.29<br>1.69-3.12)*	*0.81<br>0.70-0.93)*	1.09<br>0.97-1.23)	*1.28<br>1.13-1.46)*	*3.33<br>1.93-5.77)*	1.01<br>0.74-1.39)	1.08<br>0.85-1.36)
0.81<br>0.54-1.20)	*2.50<br>1.65-3.78)*	*0.50<br>0.34-0.72)*	1.30<br>0.73-2.32)	0.84<br>0.51-1.38)	*0.39<br>0.27-0.58)*	*0.44<br>0.32-0.59)*	Criz	*0.35<br>0.25-0.50)*	*0.48<br>0.34-0.66)*	*0.56<br>0.40-0.78)*	1.45<br>0.78-2.72)	*0.44<br>0.28-0.69)*	*0.47<br>0.32-0.69)*
2.29<br>1.77-2.96)	*7.08<br>4.34-11.5)*	*1.41<br>1.19-1.67)*	*3.69<br>2.25-6.05)*	*2.38<br>1.56-3.62)*	1.12<br>0.92-1.37)	*1.24<br>1.07-1.43)*	*2.84<br>2.02-3.98)*	Dum	*1.35<br>1.18-1.55)*	*1.59<br>1.37-1.84)*	*4.12<br>2.34-7.27)*	1.25<br>0.94-1.66)	*1.33<br>1.09-1.63)*
1.69<br>1.31-2.19)	*5.24<br>3.24-8.47)*	1.04<br>0.85-1.28)	*2.73<br>1.70-4.40)*	*1.76<br>1.16-2.66)*	0.83<br>0.66-1.05)	0.92<br>0.81-1.03)	*2.10<br>1.51-2.92)*	*0.74<br>0.65-0.85)*	Erlo	*1.18<br>1.01-1.37)*	*3.05<br>1.74-5.35)*	0.93<br>0.68-1.27)	0.99<br>0.79-1.23)
1.44<br>1.09-1.90)	*4.46<br>2.75-7.24)*	*0.89<br>0.72-1.10)*	*2.33<br>1.41-3.83)*	1.50<br>0.99-2.27)	*0.70<br>0.55-0.90)*	*0.78<br>0.68-0.89)*	*1.79<br>1.28-2.50)*	*0.63<br>0.54-0.73)*	*0.85<br>0.73-0.99)*	Gefi	*2.60<br>1.48-4.56)*	0.79<br>0.57-1.09)	0.84<br>0.67-1.06)
0.55<br>0.30-1.02)	1.72<br>0.84-3.52)	*0.34<br>0.19-0.61)*	0.90<br>0.43-1.87)	0.58<br>0.29-1.13)	*0.27<br>0.15-0.49)*	*0.30<br>0.17-0.52)*	0.69<br>0.37-1.29)	*0.24<br>0.14-0.43)*	*0.33<br>0.19-0.57)*	*0.39<br>0.22-0.68)*	Osim	*0.30<br>0.16-0.57)*	*0.32<br>0.18-0.59)*
1.82<br>1.24-2.68)	*5.65<br>3.21-9.93)*	1.12<br>0.81-1.56)	*2.94<br>1.66-5.21)*	*1.90<br>1.14-3.15)*	0.89<br>0.63-1.26)	0.99<br>0.72-1.36)	*2.26<br>1.45-3.52)*	0.80<br>0.60-1.06)	1.08<br>0.79-1.48)	1.27<br>0.92-1.74)	*3.29<br>1.75-6.20)*	Ramu	1.06<br>0.75-1.50)
1.71<br>1.25-2.36)	*5.31<br>3.15-8.94)*	1.06<br>0.82-1.37)	*2.77<br>1.64-4.68)*	*1.78<br>1.13-2.82)*	0.84<br>0.63-1.11)	0.93<br>0.74-1.17)	*2.13<br>1.45-3.13)*	*0.75<br>0.61-0.92)*	1.01<br>0.81-1.27)	1.19<br>0.94-1.50)	*3.09<br>1.71-5.61)*	0.94<br>0.66-1.33)	Vand

Drugs are reported in alphabetical order. Data in the right-upper triangle are HRs (95%CI) in the row-defining treatment compared with the column-defining treatment. HRs lower than 1 favour the row-defining treatment (the first drug in alphabetical order). HRs for the opposite comparison of PFS are in the left-lower triangle. Each comparison is shown twice in the table, once with drug A versus drug B and once with drug B versus drug A. Significant results are in italic and underscored. HR, hazard ratio; CI, confidence interval; PFS: progression-free survival. Afat, afatinib; Alec, alectinib; Beva, bevacizumab; Cabo, cabozantinib; Ceri, ceritinib; Cetu, cetuximab; Chem, chemotherapy; Criz, crizotinib; Dum, dummy; Erlo, erlotinib; Gefi, gefitinib; Osim, osimertinib; Ramu, ramucirumab; Vand, vandetanib.

Table S6. Treatment ranking probability based on overall response rate

Rank	[1]	[2]	[3]	[4]	[5]	[6]	[7]	[8]	[9]	[10]	[11]	[12]	[13]	[14]	SUCRA
Afatinib	3.40	14.21	24.69	23.67	19.88	11.92	1.54	0.39	0.17	0.07	0.04	0.01	0.01	0.00	77.82
Alectinib	6.87	19.81	24.12	21.02	15.64	8.11	2.07	0.91	0.60	0.40	0.23	0.11	0.10	0.03	79.52
Bevacizumab	0.00	0.00	0.01	0.07	0.46	2.91	11.01	18.85	23.54	21.20	12.93	6.02	2.98	0.02	36.52
Cabozantinib	62.53	9.42	5.48	4.35	4.24	4.35	1.65	1.11	0.85	0.81	0.71	0.81	1.20	2.47	87.18
Ceritinib	21.73	39.86	18.08	9.98	6.15	3.22	0.55	0.20	0.12	0.06	0.03	0.02	0.01	0.00	88.20
Cetuximab	0.00	0.01	0.04	0.19	0.73	2.84	7.55	10.23	11.97	13.81	14.33	13.18	21.48	3.63	27.07
Chemotherapy	0.00	0.00	0.00	0.00	0.00	0.04	0.39	1.93	5.76	12.96	23.12	30.43	24.12	1.24	19.20
Crizotinib	0.55	4.08	12.68	24.36	30.32	20.90	4.03	1.53	0.75	0.47	0.21	0.10	0.03	0.01	71.01
Dummy	0.00	0.00	0.00	0.00	0.00	0.00	0.00	0.00	0.00	0.01	0.08	0.97	11.10	87.83	1.02
Erlotinib	0.00	0.00	0.00	0.00	0.00	0.04	0.36	1.63	5.24	12.31	23.75	32.57	23.32	0.79	19.06
Gefitinib	0.00	0.00	0.02	0.14	0.92	5.90	21.77	25.85	21.75	14.38	7.08	1.69	0.49	0.00	42.77
Osimertinib	4.91	12.47	14.24	13.95	14.87	18.82	5.94	3.52	2.61	2.10	1.71	1.57	1.85	1.45	68.33
Ramucirumab	0.01	0.12	0.46	1.44	3.84	10.34	19.35	13.80	11.78	10.42	8.31	7.70	10.04	2.38	39.04
Vandetanib	0.00	0.02	0.18	0.83	2.95	10.61	23.79	20.05	14.86	10.99	7.48	4.82	3.28	0.14	43.26

Table S7. Treatment ranking probability based on progression-free survival

Rank	[1]	[2]	[3]	[4]	[5]	[6]	[7]	[8]	[9]	[10]	[11]	[12]	[13]	[14]	SUCRA
Afatinib	0.00	0.30	2.90	13.98	37.17	44.10	1.25	0.23	0.06	0.02	0.00	0.00	0.00	0.00	67.16
Alectinib	87.48	10.81	1.65	0.05	0.00	0.00	0.00	0.00	0.00	0.00	0.00	0.00	0.00	0.00	98.89
Bevacizumab	0.00	0.00	0.00	0.00	0.07	1.15	12.98	28.74	22.81	16.35	10.78	5.52	1.57	0.03	38.27
Cabozantinib	3.59	33.67	39.25	14.21	6.23	2.82	0.14	0.06	0.02	0.00	0.00	0.00	0.00	0.00	84.97
Ceritinib	0.03	1.96	8.31	19.51	30.25	33.60	3.36	1.40	0.68	0.39	0.25	0.16	0.07	0.04	68.42
Cetuximab	0.00	0.00	0.00	0.00	0.00	0.03	0.40	1.68	3.42	5.70	10.48	22.67	39.65	15.96	13.03
Chemotherapy	0.00	0.00	0.00	0.00	0.00	0.00	0.02	1.31	6.27	17.41	31.28	31.56	11.72	0.43	21.36
Crizotinib	0.00	5.00	21.46	42.85	21.54	8.87	0.21	0.05	0.02	0.01	0.00	0.00	0.00	0.00	76.25
Dummy	0.00	0.00	0.00	0.00	0.00	0.00	0.00	0.00	0.00	0.00	0.04	2.00	23.58	74.38	2.13
Erlotinib	0.00	0.00	0.00	0.00	0.00	0.05	1.96	17.84	30.48	28.68	15.93	4.40	0.66	0.00	34.27
Gefitinib	0.00	0.00	0.00	0.00	0.19	5.27	65.26	21.05	6.18	1.67	0.33	0.04	0.01	0.00	51.20
Osimertinib	8.89	48.27	26.42	9.40	4.38	2.27	0.19	0.08	0.04	0.02	0.02	0.01	0.00	0.00	87.63
Ramucirumab	0.00	0.00	0.00	0.00	0.12	1.08	7.10	10.70	11.26	11.25	13.61	19.87	16.53	8.46	24.77
Vandetanib	0.00	0.00	0.00	0.00	0.04	0.76	7.13	16.87	18.76	18.50	17.28	13.76	6.21	0.69	31.61

eReference. List of studies included in the network meta-analysis

1. Aisner, J.; Manola, J.B.; Dakhil, S.R.; Stella, P.J.; Sovak, M.A.; Schiller, J.H. Vandetanib plus chemotherapy for induction followed by vandetanib or placebo as maintenance for patients with advanced non-small-cell lung cancer: a randomized phase 2 PrECOG study (PrE0501). *Journal of thoracic oncology* **2013**, *8*, 1075-1083, doi:10.1097/JTO.0b013e3182937317.
2. Arnold, A.M.; Seymour, L.; Smylie, M.; Ding, K.; Ung, Y.; Findlay, B.; Lee, C.W.; Djurfeldt, M.; Whitehead, M.; Ellis, P., et al. Phase II study of vandetanib or placebo in small-cell lung cancer patients after complete or partial response to induction chemotherapy with or without radiation therapy: National Cancer Institute of Canada Clinical Trials Group Study BR.20. *Journal of Clinical Oncology* **2007**, *25*, 4278-4284, doi:10.1200/JCO.2007.12.3083.
3. Auliac, J.B.; Chouaid, C.; Greillier, L.; Greiller, L.; Monnet, I.; Le Caer, H.; Falchero, L.; Corre, R.; Descourt, R.; Bota, S., et al. Randomized open-label non-comparative multicenter phase II trial of sequential erlotinib and docetaxel versus docetaxel alone in patients with non-small-cell lung cancer after failure of first-line chemotherapy: GFPC 10.02 study. *Lung cancer (amsterdam, netherlands)* **2014**, *85*, 415-419, doi:10.1016/j.lungcan.2014.07.006.
4. Boutsikou, E.; Kontakiotis, T.; Zarogoulidis, P.; Darwiche, K.; Eleptheriadou, E.; Porpodis, K.; Galaktidou, G.; Sakkas, L.; Hohenforst-Schmidt, W.; Tsakiridis, K., et al. Docetaxel-carboplatin in combination with erlotinib and/or bevacizumab in patients with non-small cell lung cancer. *Oncotargets and therapy* **2013**, *6*, 125-134, doi:10.2147/OTT.S42245.
5. Brahmer, J.R.; Dahlberg, S.E.; Gray, R.J.; Schiller, J.H.; Perry, M.C.; Sandler, A.; Johnson, D.H. Sex differences in outcome with bevacizumab therapy: Analysis of patients with advanced-stage non-small cell lung cancer treated with or without bevacizumab in combination with paclitaxel and carboplatin in the eastern cooperative oncology group trial 4599. *Journal of Thoracic Oncology* **2011**, *6*, 103-108, doi:10.1097/JTO.0b013e3181fa8efd.
6. Butts, C.A.; Bodkin, D.; Middleman, E.L.; Englund, C.W.; Ellison, D.; Alam, Y.; Kreisman, H.; Graze, P.; Maher, J.; Ross, H.J., et al. Randomized phase II study of gemcitabine plus cisplatin or carboplatin [corrected], with or without cetuximab, as first-line therapy for patients with advanced or metastatic non small-cell lung cancer. *J Clin Oncol* **2007**, *25*, 5777-5784, doi:10.1200/JCO.2007.13.0856.
7. Bylicki, O.; Ferlay, C.; Chouaid, C.; Lavolé, A.; Barlési, F.; Dubos, C.; Westeel, V.; Créquit, J.; Corre, R.; Vergnenègre, A., et al. Efficacy of pemetrexed as second-line therapy in advanced NSCLC after either treatment-free interval or maintenance therapy with gemcitabine or erlotinib in IFCT-GFPC 05-02 phase III study. *Journal of thoracic oncology* **2013**, *8*, 906-914, doi:10.1097/JTO.0b013e31828cb505.
8. Cappuzzo, F.; Ciuleanu, T.; Stelmakh, L.; Cicenas, S.; Szczésna, A.; Juhász, E.; Esteban, E.; Molinier, O.; Brugger, W.; Melezínek, I., et al. Erlotinib as maintenance treatment in advanced non-small-cell lung cancer: A multicentre, randomised, placebo-controlled phase 3 study. *The Lancet Oncology* **2010**, *11*, 521-529, doi:10.1016/S1470-2045(10)70112-1.
9. Chen, Y.M.; Tsai, C.M.; Fan, W.C.; Shih, J.F.; Liu, S.H.; Wu, C.H.; Chou, T.Y.; Lee, Y.C.; Perng, R.P.; Whang-Peng, J. Phase II randomized trial of erlotinib or vinorelbine in chemonaive, advanced, non-small cell lung cancer patients aged 70 years or older. *Journal of thoracic oncology : official publication of the International Association for the Study of Lung Cancer* **2012**, *7*, 412-418, doi:10.1097/JTO.0b013e31823a39e8.
10. Choi, Y.J.; Lee, D.H.; Choi, C.M.; Lee, J.S.; Lee, S.J.; Ahn, J.H.; Kim, S.W. Randomized phase II study of paclitaxel/carboplatin intercalated with gefitinib compared to paclitaxel/carboplatin alone for chemotherapy-naive non-small cell lung cancer in a clinically selected population excluding patients with non-smoking adenocarcinoma or mutated EGFR. *BMC cancer* **2015**, *15*, doi:10.1186/s12885-015-1714-y.
11. Ciuleanu, T.; Stelmakh, L.; Cicenas, S.; Miliauskas, S.; Grigorescu, A.C.; Hillenbach, C.; Johannsdottir, H.K.; Klughammer, B.; Gonzalez, E.E. Efficacy and safety of erlotinib versus chemotherapy in second-line treatment of patients with advanced, non-small-cell lung cancer with poor prognosis (TITAN): a randomised multicentre, open-label, phase 3 study. *The lancet. Oncology* **2012**, *13*, 300-308, doi:10.1016/S1470-2045(11)70385-0.
12. Ciuleanu, T.; Tsai, C.M.; Tsao, C.J.; Milanowski, J.; Amoroso, D.; Heo, D.S.; Groen, H.J.; Szczesna, A.; Chung, C.Y.; Chao, T.Y., et al. A phase II study of erlotinib in combination with bevacizumab versus chemotherapy plus bevacizumab in the first-line treatment of advanced non-squamous non-small cell lung cancer. *Lung cancer (amsterdam, netherlands)* **2013**, *82*, 276-281, doi:10.1016/j.lungcan.2013.08.002.
13. Crino, L.; Cappuzzo, F.; Zatloukal, P.; Reck, M.; Pesek, M.; Thompson, J.C.; Ford, H.E.; Hirsch, F.R.; Varella-Garcia, M.; Ghiorghiu, S., et al. Gefitinib versus vinorelbine in chemotherapy-naive elderly

patients with advanced non-small-cell lung cancer (INVITE): a randomized, phase II study. *J Clin Oncol* **2008**, *26*, 4253-4260, doi:10.1200/jco.2007.15.0672.
14. Cufer, T.; Vrdoljak, E.; Gaafar, R.; Erensoy, I.; Pemberton, K. Phase II, open-label, randomized study (SIGN) of single-agent gefitinib (IRESSA) or docetaxel as second-line therapy in patients with advanced (stage IIIb or IV) non-small-cell lung cancer. *Anti-cancer drugs* **2006**, *17*, 401-409, doi:10.1097/01.cad.0000203381.99490.ab.
15. Dai, H.; Xu, L.; Xia, C.; Chen, W. A randomized clinical study of Gefitinib and pemetrexed as second line therapy for advanced non-squamous non-small cell lung cancer. *Zhongguo fei ai za zhi [Chinese journal of lung cancer]* **2013**, *16*, 405-410, doi:10.3779/j.issn.1009-3419.2013.08.03.
16. de Boer, R.H.; Arrieta, Ó.; Yang, C.H.; Gottfried, M.; Chan, V.; Raats, J.; de Marinis, F.; Abratt, R.P.; Wolf, J.; Blackhall, F.H., et al. Vandetanib plus pemetrexed for the second-line treatment of advanced non-small-cell lung cancer: a randomized, double-blind phase III trial. *Journal of clinical oncology* **2011**, *29*, 1067-1074, doi:10.1200/JCO.2010.29.5717.
17. Dittrich, C.; Papai-Szekely, Z.; Vinolas, N.; Sederholm, C.; Hartmann, J.T.; Behringer, D.; Kazeem, G.; Desaiah, D.; Leschinger, M.I.; von Pawel, J. A randomised phase II study of pemetrexed versus pemetrexed+erlotinib as second-line treatment for locally advanced or metastatic non-squamous non-small cell lung cancer. *European journal of cancer (oxford, england : 1990)* **2014**, *50*, 1571-1580, doi:10.1016/j.ejca.2014.03.007.
18. Doebele, R.C.; Spigel, D.; Tehfe, M.; Thomas, S.; Reck, M.; Verma, S.; Eakle, J.; Bustin, F.; Goldschmidt, J.; Cao, D., et al. Phase 2, randomized, open-label study of ramucirumab in combination with first-line pemetrexed and platinum chemotherapy in patients with nonsquamous, advanced/metastatic non-small cell lung cancer. *Cancer* **2015**, *121*, 883-892, doi:10.1002/cncr.29132.
19. Felip, E.; Soria, J.C.; Cobo, M.; Lu, S.; Syrigos, K.; Lee, K.H.; Göker, E.; Georgoulias, V.; Li, W.; Guclu, S., et al. Second-line afatinib versus erlotinib for patients with squamous cell carcinoma of the lung (LUX-lung 8): Analysis of tumor and serum biomarkers. *Journal of Thoracic Oncology* **2017**, *12*, S1186-S1187.
20. Fiala, O.; Pesek, M.; Finek, J.; Krejci, J.; Bortlicek, Z.; Benesova, L.; Minarik, M. Second-line treatment of advanced NSCLC: comparison of efficacy of erlotinib and chemotherapy. *Neoplasma* **2013**, *60*, 129-134, doi:10.4149/neo_2013_017.
21. Gaafar, R.M.; Surmont, V.F.; Scagliotti, G.V.; Van Klaveren, R.J.; Papamichael, D.; Welch, J.J.; Hasan, B.; Torri, V.; van Meerbeeck, J.P. A double-blind, randomised, placebo-controlled phase III intergroup study of gefitinib in patients with advanced NSCLC, non-progressing after first line platinum-based chemotherapy (EORTC 08021/ILCP 01/03). *European journal of cancer (oxford, england : 1990)* **2011**, *47*, 2331-2340, doi:10.1016/j.ejca.2011.06.045.
22. Garassino, M.C.; Martelli, O.; Broggini, M.; Farina, G.; Veronese, S.; Rulli, E.; Bianchi, F.; Bettini, A.; Longo, F.; Moscetti, L., et al. Erlotinib versus docetaxel as second-line treatment of patients with advanced non-small-cell lung cancer and wild-type EGFR tumours (TAILOR): a randomised controlled trial. *The lancet. Oncology* **2013**, *14*, 981-988, doi:10.1016/S1470-2045(13)70310-3.
23. Gatzemeier, U.; Pluzanska, A.; Szczesna, A.; Kaukel, E.; Roubec, J.; De Rosa, F.; Milanowski, J.; Karnicka-Mlodkowski, H.; Pesek, M.; Serwatowski, P., et al. Phase III study of erlotinib in combination with cisplatin and gemcitabine in advanced non-small-cell lung cancer: the Tarceva Lung Cancer Investigation Trial. *Journal of clinical oncology* **2007**, *25*, 1545-1552, doi:10.1200/JCO.2005.05.1474.
24. Gautschi, O.; Rothschild, S.I.; Li, Q.; Matter-Walstra, K.; Zippelius, A.; Betticher, D.C.; Fruh, M.; Stahel, R.A.; Cathomas, R.; Rauch, D., et al. Bevacizumab plus pemetrexed versus pemetrexed alone as maintenance therapy for patients with advanced nonsquamous non-small-cell lung cancer: Update from the Swiss group for clinical cancer research (SAKK) 19/09 trial. *Clinical lung cancer* **2017**, *18*, 303-309, doi:10.1016/j.cllc.2016.11.007.
25. Goss, G.; Ferry, D.; Wierzbicki, R.; Laurie, S.A.; Thompson, J.; Biesma, B.; Hirsch, F.R.; Varella-Garcia, M.; Duffield, E.; Ataman, O.U., et al. Randomized phase II study of gefitinib compared with placebo in chemotherapy-naive patients with advanced non-small-cell lung cancer and poor performance status. *Journal of Clinical Oncology* **2009**, *27*, 2253-2260, doi:10.1200/JCO.2008.18.4408.
26. Gregorc, V.; Lazzari, C.; Novello, S.; Barni, S.; Aieta, M.; Grossi, F.; De Pas, T.; De Marinis, F.; Mencoboni, M.; Bearz, A., et al. Randomized proteomic stratified phase iii study of second line erlotinib (E) versus chemotherapy (CT) in patients with inoperable non-small cell lung cancer (PROSE): secondary endpoint analysis. *Journal of thoracic oncology.* **2013**, *8*, S134, doi:10.1097/01.JTO.0000438438.14562.c8.
27. Gridelli, C.; Novello, S.; Zilembo, N.; Luciani, A.; Favaretto, A.G.; De Marinis, F.; Genestreti, G.; Crinò, L.; Grossi, F.; Caffo, O., et al. Phase II randomized study of vandetanib plus gemcitabine or gemcitabine

plus placebo as first-line treatment of advanced non-small-cell lung cancer in elderly patients. *Journal of thoracic oncology* **2014**, *9*, 733-737, doi:10.1097/JTO.0000000000000120.
28. Gupta, K.; Joshi, A.; Noronha, V.; Parthiban, S.K.; Patil, V.; Janu, A.; Kaushal, R.; Prabhash, K. Maintenance therapy using tyrosine kinase inhibitor (erlotinib) or pemetrexed in metastatic/locally advanced in EGFR mutation-negative lung cancer: comparison of results. *Journal of thoracic oncology. Conference: 8th european lung cancer congress, ELCC 2018. Switzerland* **2018**, *13*, S91.
29. Halmos, B.; Pennell, N.A.; Fu, P.; Saad, S.; Gadgeel, S.; Otterson, G.A.; Mekhail, T.; Snell, M.; Kuebler, J.P.; Sharma, N., et al. Randomized Phase II Trial of Erlotinib Beyond Progression in Advanced Erlotinib-Responsive Non-Small Cell Lung Cancer. *Oncologist* **2015**, *20*, 1298-1303, doi:10.1634/theoncologist.2015-0136.
30. Heigener, D.F.; Deppermann, K.M.; Pawel, J.V.; Fischer, J.R.; Kortsik, C.; Bohnet, S.; Eiff, M.V.; Koester, W.; Thomas, M.; Schnabel, P.A., et al. Open, randomized, multi-center phase II study comparing efficacy and tolerability of Erlotinib vs. Carboplatin/Vinorelbin in elderly patients (>70 years of age) with untreated non-small cell lung cancer. *Lung cancer (amsterdam, netherlands)* **2014**, *84*, 62-66, doi:10.1016/j.lungcan.2014.01.024.
31. Herbst, R.S.; Ansari, R.; Bustin, F.; Flynn, P.; Hart, L.; Otterson, G.A.; Vlahovic, G.; Soh, C.H.; O'Connor, P.; Hainsworth, J. Efficacy of bevacizumab plus erlotinib versus erlotinib alone in advanced non-small-cell lung cancer after failure of standard first-line chemotherapy (BeTa): a double-blind, placebo-controlled, phase 3 trial. *Lancet (london, england)* **2011**, *377*, 1846-1854, doi:10.1016/S0140-6736(11)60545-X.
32. Herbst, R.S.; O'Neill, V.J.; Fehrenbacher, L.; Belani, C.P.; Bonomi, P.D.; Hart, L.; Melnyk, O.; Ramies, D.; Lin, M.; Sandler, A. Phase II study of efficacy and safety of bevacizumab in combination with chemotherapy or erlotinib compared with chemotherapy alone for treatment of recurrent or refractory non-small-cell lung cancer. *Journal of Clinical Oncology* **2007**, *25*, 4743-4750, doi:10.1200/JCO.2007.12.3026.
33. Herbst, R.S.; Redman, M.W.; Kim, E.S.; Semrad, T.J.; Bazhenova, L.; Masters, G.; Oettel, K.; Guaglianone, P.; Reynolds, C.; Karnad, A., et al. Cetuximab plus carboplatin and paclitaxel with or without bevacizumab versus carboplatin and paclitaxel with or without bevacizumab in advanced NSCLC (SWOG S0819): a randomised, phase 3 study. *Lancet oncology* **2017**, *(no pagination)*, doi:10.1016/S1470-2045%2817%2930694-0.
34. Herbst, R.S.; Sun, Y.; Eberhardt, W.E.; Germonpré, P.; Saijo, N.; Zhou, C.; Wang, J.; Li, L.; Kabbinavar, F.; Ichinose, Y., et al. Vandetanib plus docetaxel versus docetaxel as second-line treatment for patients with advanced non-small-cell lung cancer (ZODIAC): a double-blind, randomised, phase 3 trial. *The lancet. Oncology* **2010**, *11*, 619-626, doi:10.1016/S1470-2045(10)70132-7.
35. Heymach, J.V.; Johnson, B.E.; Prager, D.; Csada, E.; Roubec, J.; Pesek, M.; Spasova, I.; Belani, C.P.; Bodrogi, I.; Gadgeel, S., et al. Randomized, placebo-controlled phase II study of vandetanib plus docetaxel in previously treated non small-cell lung cancer. *J Clin Oncol* **2007**, *25*, 4270-4277, doi:10.1200/jco.2006.10.5122.
36. Hida, T.; Nokihara, H.; Kondo, M.; Kim, Y.H.; Azuma, K.; Seto, T.; Takiguchi, Y.; Nishio, M.; Yoshioka, H.; Imamura, F., et al. Alectinib versus crizotinib in patients with ALK-positive non-small-cell lung cancer (J-ALEX): an open-label, randomised phase 3 trial. *Lancet* **2017**, *(no pagination)*, doi:10.1016/S0140-6736%2817%2930565-2.
37. Hong, J.; Kyung, S.Y.; Lee, S.P.; Park, J.W.; Jung, S.H.; Lee, J.I.; Park, S.H.; Sym, S.J.; Park, J.; Cho, E.K., et al. Pemetrexed versus gefitinib versus erlotinib in previously treated patients with non-small cell lung cancer. *The Korean journal of internal medicine* **2010**, *25*, 294-300, doi:10.3904/kjim.2010.25.3.294.
38. Hosomi, Y.; Yoh, K.; Kasahara, K.; Yamada, K.; Takahashi, T.; Tanaka, K.; Hida, T.; Yoshioka, H.; Kato, T.; Takeda, K., et al. Docetaxel + ramucirumab (DR) versus docetaxel + placebo (D) as second-line treatment for advanced non-small cell lung cancer (NSCLC): a randomized, phase II, double-blind, multicenter trial in Japan. *Journal of clinical oncology* **2015**, *33*.
39. Inoue, A.; Kobayashi, K.; Maemondo, M.; Sugawara, S.; Oizumi, S.; Isobe, H.; Gemma, A.; Harada, M.; Yoshizawa, H.; Kinoshita, I., et al. Updated overall survival results from a randomized phase III trial comparing gefitinib with carboplatin-paclitaxel for chemo-naive non-small cell lung cancer with sensitive EGFR gene mutations (NEJ002). *Annals of oncology : official journal of the European Society for Medical Oncology* **2013**, *24*, 54-59, doi:10.1093/annonc/mds214.
40. Johnson, D.H.; Fehrenbacher, L.; Novotny, W.F.; Herbst, R.S.; Nemunaitis, J.J.; Jablons, D.M.; Langer, C.J.; DeVore, R.F.; Gaudreault, J.; Damico, L.A., et al. Randomized phase II trial comparing bevacizumab plus carboplatin and paclitaxel with carboplatin and paclitaxel alone in previously untreated locally advanced or metastatic non-small-cell lung cancer. *Journal of clinical oncology* **2004**, *22*, 2184-2191, doi:10.1200/JCO.2004.11.022.

41. Kabbinavar, F.; Fehrenbacher, L.; Hainsworth, J.; Kasubhai, S.; Kressel, B.; Marsland, T.; Patel, T.; Rubin, M.; White, L.; Yang, J.C., et al. Biomarker analyses from a randomized, placebo-controlled, phase IIIb trial comparing bevacizumab with or without erlotinib as maintenance therapy for the treatment of advanced non-small-cell lung cancer (ATLAS). *Journal of thoracic oncology* **2014**, *9*, 1411-1417, doi:10.1097/JTO.0000000000000274.
42. Karayama, M.; Inui, N.; Fujisawa, T.; Enomoto, N.; Nakamura, Y.; Kuroishi, S.; Yokomura, K.; Koshimizu, N.; Sato, M.; Toyoshima, M., et al. Maintenance therapy with pemetrexed and bevacizumab versus pemetrexed monotherapy after induction therapy with carboplatin, pemetrexed, and bevacizumab in patients with advanced non-squamous non small cell lung cancer. *European journal of cancer (oxford, england : 1990)* **2016**, *58*, 30-37, doi:10.1016/j.ejca.2016.01.013.
43. Kawaguchi, T.; Ando, M.; Asami, K.; Okano, Y.; Fukuda, M.; Nakagawa, H.; Ibata, H.; Kozuki, T.; Endo, T.; Tamura, A., et al. Randomized phase III trial of erlotinib versus docetaxel as second- or third-line therapy in patients with advanced non-small-cell lung cancer: docetaxel and Erlotinib Lung Cancer Trial (DELTA). *Journal of clinical oncology* **2014**, *32*, 1902-1908, doi:10.1200/JCO.2013.52.4694.
44. Kelly, K.; Altorki, N.K.; Eberhardt, W.E.; O'Brien, M.E.; Spigel, D.R.; Crinò, L.; Tsai, C.M.; Kim, J.H.; Cho, E.K.; Hoffman, P.C., et al. Adjuvant erlotinib versus placebo in patients with stage IB-IIIA non-small-cell lung cancer (RADIANT): a randomized, double-blind, phase III trial. *Journal of clinical oncology* **2015**, *33*, 4007-4014, doi:10.1200/JCO.2015.61.8918.
45. Kelly, K.; Azzoli, C.G.; Zatloukal, P.; Albert, I.; Jiang, P.Y.; Bodkin, D.; Pereira, J.R.; Juhasz, E.; Iannotti, N.O.; Weems, G., et al. Randomized phase 2b study of pralatrexate versus erlotinib in patients with stage IIIB/IV non-small-cell lung cancer (NSCLC) after failure of prior platinum-based therapy. *Journal of thoracic oncology : official publication of the International Association for the Study of Lung Cancer* **2012**, *7*, 1041-1048, doi:10.1097/JTO.0b013e31824cc66c.
46. Kelly, K.; Chansky, K.; Gaspar, L.E.; Albain, K.S.; Jett, J.; Ung, Y.C.; Lau, D.H.; Crowley, J.J.; Gandara, D.R. Phase III trial of maintenance gefitinib or placebo after concurrent chemoradiotherapy and docetaxel consolidation in inoperable stage III non-small-cell lung cancer: SWOG S0023. *Journal of clinical oncology* **2008**, *26*, 2450-2456, doi:10.1200/JCO.2007.14.4824.
47. Kim, E.S.; Hirsh, V.; Mok, T.; Socinski, M.A.; Gervais, R.; Wu, Y.L.; Li, L.Y.; Watkins, C.L.; Sellers, M.V.; Lowe, E.S., et al. Gefitinib versus docetaxel in previously treated non-small-cell lung cancer (INTEREST): a randomised phase III trial. *Lancet (london, england)* **2008**, *372*, 1809-1818, doi:10.1016/S0140-6736(08)61758-4.
48. Kim, E.S.; Neubauer, M.; Cohn, A.; Schwartzberg, L.; Garbo, L.; Caton, J.; Robert, F.; Reynolds, C.; Katz, T.; Chittoor, S., et al. Docetaxel or pemetrexed with or without cetuximab in recurrent or progressive non-small-cell lung cancer after platinum-based therapy: a phase 3, open-label, randomised trial. *The lancet. Oncology* **2013**, *14*, 1326-1336, doi:10.1016/S1470-2045(13)70473-X.
49. Kim, S.T.; Uhm, J.E.; Lee, J.; Sun, J.M.; Sohn, I.; Kim, S.W.; Jung, S.H.; Park, Y.H.; Ahn, J.S.; Park, K., et al. Randomized phase II study of gefitinib versus erlotinib in patients with advanced non-small cell lung cancer who failed previous chemotherapy. *Lung cancer (amsterdam, netherlands)* **2012**, *75*, 82-88, doi:10.1016/j.lungcan.2011.05.022.
50. Kim, Y.S.; Cho, E.K.; Woo, H.S.; Hong, J.; Ahn, H.K.; Park, I.; Sym, S.J.; Kyung, S.Y.; Kang, S.M.; Park, J.W., et al. Randomized phase II study of pemetrexed versus gefitinib in previously treated patients with advanced non-small cell lung cancer. *Cancer research and treatment* **2016**, *48*, 80-87, doi:10.4143/crt.2014.307.
51. Lee, D.H.; Lee, J.S.; Kim, S.W.; Rodrigues-Pereira, J.; Han, B.; Song, X.Q.; Wang, J.; Kim, H.K.; Sahoo, T.P.; Digumarti, R., et al. Three-arm randomised controlled phase 2 study comparing pemetrexed and erlotinib to either pemetrexed or erlotinib alone as second-line treatment for never-smokers with non-squamous non-small cell lung cancer. *Eur J Cancer* **2013**, *49*, 3111-3121, doi:10.1016/j.ejca.2013.06.035.
52. Lee, D.H.; Park, K.; Kim, J.H.; Lee, J.S.; Shin, S.W.; Kang, J.H.; Ahn, M.J.; Ahn, J.S.; Suh, C.; Kim, S.W. Randomized phase III trial of gefitinib versus docetaxel in non-small cell lung cancer patients who have previously received platinum-based chemotherapy. *Clinical Cancer Research* **2010**, *16*, 1307-1314, doi:10.1158/1078-0432.CCR-09-1903.
53. Lee, J.S.; Lee, Y.J.; Kim, H.Y.; Nam, B.H.; Lee, G.K.; Kim, H.T.; Han, J.Y. Randomized phase II trial of intercalated gefitinib (G) and pemetrexed/cisplatin (Pem/Cis) for never-smokers with chemo-naive stage IIIB/IV lung adenocarcinoma (LADC). *Journal of Clinical Oncology* **2016**, *34*.
54. Lee, S.M.; Khan, I.; Upadhyay, S.; Lewanski, C.; Falk, S.; Skailes, G.; Marshall, E.; Woll, P.J.; Hatton, M.; Lal, R., et al. First-line erlotinib in patients with advanced non-small-cell lung cancer unsuitable for

chemotherapy (TOPICAL): a double-blind, placebo-controlled, phase 3 trial. *The lancet. Oncology* **2012**, *13*, 1161-1170, doi:10.1016/S1470-2045(12)70412-6.
55. Li, N.; Ou, W.; Ye, X.; Sun, H.B.; Zhang, L.; Fang, Q.; Zhang, S.L.; Wang, B.X.; Wang, S.Y. Pemetrexed-carboplatin adjuvant chemotherapy with or without gefitinib in resected stage IIIA-N2 non-small cell lung cancer harbouring EGFR mutations: a randomized, phase II study. *Annals of surgical oncology* **2014**, *21*, 2091-2096, doi:10.1245/s10434-014-3586-9.
56. Lilenbaum, R.; Axelrod, R.; Thomas, S.; Dowlati, A.; Seigel, L.; Albert, D.; Witt, K.; Botkin, D. Randomized phase II trial of erlotinib or standard chemotherapy in patients with advanced non-small-cell lung cancer and a performance status of 2. *Journal of clinical oncology* **2008**, *26*, 863-869, doi:10.1200/JCO.2007.13.2720.
57. Lu, S.; Mok, T.; Lu, Y.; Zhou, J.; Shi, Y.; Sriuranpong, V.; Ho, J.C.M.; Ong, C.K.; Tsai, C.M.; Chung, C.H., et al. Phase 3 study of first-line crizotinib vs pemetrexed-cisplatin/carboplatin (PCC) in East Asian patients (pts) with ALK+ advanced non-squamous non-small cell lung cancer (NSCLC). *Journal of Clinical Oncology* **2016**, *34*.
58. Lynch, T.J.; Patel, T.; Dreisbach, L.; McCleod, M.; Heim, W.J.; Hermann, R.C.; Paschold, E.; Iannotti, N.O.; Dakhil, S.; Gorton, S., et al. Cetuximab and first-line taxane/carboplatin chemotherapy in advanced non-small-cell lung cancer: results of the randomized multicenter phase III trial BMS099. *J Clin Oncol* **2010**, *28*, 911-917, doi:10.1200/jco.2009.21.9618.
59. Maemondo, M.; Inoue, A.; Kobayashi, K.; Sugawara, S.; Oizumi, S.; Isobe, H.; Gemma, A.; Harada, M.; Yoshizawa, H.; Kinoshita, I., et al. Gefitinib or chemotherapy for non-small-cell lung cancer with mutated EGFR. *New England Journal of Medicine* **2010**, *362*, 2380-2388, doi:10.1056/NEJMoa0909530.
60. Maruyama, R.; Nishiwaki, Y.; Tamura, T.; Yamamoto, N.; Tsuboi, M.; Nakagawa, K.; Shinkai, T.; Negoro, S.; Imamura, F.; Eguchi, K., et al. Phase III study, V-15-32, of gefitinib versus docetaxel in previously treated Japanese patients with non-small-cell lung cancer. *Journal of Clinical Oncology* **2008**, *26*, 4244-4252, doi:10.1200/JCO.2007.15.0185.
61. Michael, M.; White, S.C.; Abdi, E.; Nott, L.; Clingan, P.; Zimet, A.; Button, P.; Gregory, D.; Solomon, B.; Dobrovic, A., et al. Multicenter randomized, open-label phase II trial of sequential erlotinib and gemcitabine compared with gemcitabine monotherapy as first-line therapy in elderly or ECOG PS two patients with advanced NSCLC. *Asia-pacific journal of clinical oncology* **2015**, *11*, 4-14, doi:10.1111/ajco.12178.
62. Miller, V.A.; Hirsh, V.; Cadranel, J.; Chen, Y.M.; Park, K.; Kim, S.W.; Zhou, C.; Su, W.C.; Wang, M.; Sun, Y., et al. Afatinib versus placebo for patients with advanced, metastatic non-small-cell lung cancer after failure of erlotinib, gefitinib, or both, and one or two lines of chemotherapy (LUX-Lung 1): a phase 2b/3 randomised trial. *The lancet. Oncology* **2012**, *13*, 528-538, doi:10.1016/S1470-2045(12)70087-6.
63. Mitsudomi, T.; Morita, S.; Yatabe, Y.; Negoro, S.; Okamoto, I.; Tsurutani, J.; Seto, T.; Satouchi, M.; Tada, H.; Hirashima, T., et al. Gefitinib versus cisplatin plus docetaxel in patients with non-small-cell lung cancer harbouring mutations of the epidermal growth factor receptor (WJTOG3405): an open label, randomised phase 3 trial. *The Lancet Oncology* **2010**, *11*, 121-128, doi:10.1016/S1470-2045(09)70364-X.
64. Mok, T.S.; Wu, Y.L.; Ahn, M.J.; Garassino, M.C.; Kim, H.R.; Ramalingam, S.S.; Shepherd, F.A.; He, Y.; Akamatsu, H.; Theelen, W., et al. Osimertinib or platinum-pemetrexed in EGFR T790M-Positive lung cancer. *New england journal of medicine* **2017**, *376*, 629-640, doi:10.1056/NEJMoa1612674.
65. Mok, T.S.; Wu, Y.L.; Thongprasert, S.; Yang, C.H.; Chu, D.T.; Saijo, N.; Sunpaweravong, P.; Han, B.; Margono, B.; Ichinose, Y., et al. Gefitinib or carboplatin-paclitaxel in pulmonary adenocarcinoma. *New England Journal of Medicine* **2009**, *361*, 947-957, doi:10.1056/NEJMoa0810699.
66. Mok, T.S.K.; Wu, Y.L.; Yu, C.J.; Zhou, C.; Chen, Y.M.; Zhang, L.; Ignacio, J.; Liao, M.; Srimuninnimit, V.; Boyer, M.J., et al. Randomized, placebo-controlled, phase II study of sequential erlotinib and chemotherapy as first-line treatment for advanced non-small-cell lung cancer. *Journal of Clinical Oncology* **2009**, *27*, 5080-5087, doi:10.1200/JCO.2008.21.5541.
67. Morère, J.F.; Bréchot, J.M.; Westeel, V.; Gounant, V.; Lebeau, B.; Vaylet, F.; Barlési, F.; Urban, T.; Souquet, P.J.; Debieuvre, D., et al. Randomized phase II trial of gefitinib or gemcitabine or docetaxel chemotherapy in patients with advanced non-small-cell lung cancer and a performance status of 2 or 3 (IFCT-0301 study). *Lung cancer (amsterdam, netherlands)* **2010**, *70*, 301-307, doi:10.1016/j.lungcan.2010.03.003.
68. Natale, R.B.; Bodkin, D.; Govindan, R.; Sleckman, B.G.; Rizvi, N.A.; Capó, A.; Germonpré, P.; Eberhardt, W.E.E.; Stockman, P.K.; Kennedy, S.J., et al. Vandetanib versus gefitinib in patients with advanced non-small-cell lung cancer: Results from a two-part, double-blind, randomized phase II study. *Journal of Clinical Oncology* **2009**, *27*, 2523-2529, doi:10.1200/JCO.2008.18.6015.

69. Neal, J.W.; Dahlberg, S.E.; Wakelee, H.A.; Aisner, S.C.; Bowden, M.; Carbone, D.P.; Ramalingam, S.S. Cabozantinib (C), erlotinib (E) or the combination (E+C) as second-or third-line therapy in patients with EGFR wild-type (wt) non-small cell lung cancer (NSCLC): a randomized phase 2 trial of the ECOGACRIN Cancer Research Group (E1512). *Journal of clinical oncology* **2015**, *33*.
70. Neal, J.W.; Dahlberg, S.E.; Wakelee, H.A.; Aisner, S.C.; Bowden, M.; Huang, Y.; Carbone, D.P.; Gerstner, G.J.; Lerner, R.E.; Rubin, J.L., et al. Erlotinib, cabozantinib, or erlotinib plus cabozantinib as second-line or third-line treatment of patients with EGFR wild-type advanced non-small-cell lung cancer (ECOG-ACRIN 1512): a randomised, controlled, open-label, multicentre, phase 2 trial. *Lancet oncology* **2016**, *17*, 1661-1671, doi:10.1016/S1470-2045%2816%2930561-7.
71. Niho, S.; Kunitoh, H.; Nokihara, H.; Horai, T.; Ichinose, Y.; Hida, T.; Yamamoto, N.; Kawahara, M.; Shinkai, T.; Nakagawa, K., et al. Randomized phase II study of first-line carboplatin-paclitaxel with or without bevacizumab in Japanese patients with advanced non-squamous non-small-cell lung cancer. *Lung cancer (Amsterdam, Netherlands)* **2012**, *76*, 362-367, doi:10.1016/j.lungcan.2011.12.005.
72. Perol, M.; Chouaid, C.; Perol, D.; Barlesi, F.; Gervais, R.; Westeel, V.; Crequit, J.; Lena, H.; Vergnenegre, A.; Zalcman, G., et al. Randomized, phase III study of gemcitabine or erlotinib maintenance therapy versus observation,with predefined second-line treatment, after cisplatin-gemcitabine induction chemotherapy in advanced non-small-cell lung cancer. *Journal of clinical oncology* **2012**, *30*, 3516-3524, doi:10.1200/JCO.2011.39.9782.
73. Peters, S.; Camidge, D.R.; Shaw, A.T.; Gadgeel, S.; Ahn, J.S.; Kim, D.W.; Ou, S.I.; Pérol, M.; Dziadziuszko, R.; Rosell, R., et al. Alectinib versus crizotinib in untreated ALK-positive non-small-cell lung cancer. *New England journal of medicine* **2017**, *377*, 829-838, doi:10.1056/NEJMoa1704795.
74. Pirker, R.; Pereira, J.R.; Szczesna, A.; von Pawel, J.; Krzakowski, M.; Ramlau, R.; Vynnychenko, I.; Park, K.; Yu, C.T.; Ganul, V., et al. Cetuximab plus chemotherapy in patients with advanced non-small-cell lung cancer (FLEX): an open-label randomised phase III trial. *Lancet (london, england)* **2009**, *373*, 1525-1531, doi:10.1016/S0140-6736(09)60569-9.
75. Prabhash, K.; Patil, V.; Joshi, A.; Noronha, V.; Chougule, A.; Mahajan, A.; Janu, A.; Goud, S.; More, S.; Kaushal, R., et al. Pemetrexed versus gefitinib in EGFR mutation positive lung cancer: Results of a phase 3 study from India. *Journal of Thoracic Oncology* **2017**, *12*, S1246.
76. Pujol, J.L.; Lavole, A.; Quoix, E.; Molinier, O.; Souquet, P.J.; Barlesi, F.; Le Caer, H.; Moro-Sibilot, D.; Fournel, P.; Oster, J.P., et al. Randomized phase II-III study of bevacizumab in combination with chemotherapy in previously untreated extensive small-cell lung cancer: results from the IFCT-0802 trialdagger. *Annals of oncology : official journal of the European Society for Medical Oncology* **2015**, *26*, 908-914, doi:10.1093/annonc/mdv065.
77. Reck, M.; Garon, E.B.; Paz-Ares, L.; Ponce, S.; Jaime, J.C.; Juan, O.; Nadal, E.; Kiura, K.; Widau, R.C.; He, S., et al. Randomized, double-blind phase Ib/III study of erlotinib with ramucirumab or placebo in previously untreated EGFR-mutant metastatic non-small-cell lung cancer (RELAY): phase Ib results. *Clinical lung cancer* **2018**, *(no pagination)*, doi:10.1016/j.cllc.2017.11.003.
78. Reck, M.; von Pawel, J.; Zatloukal, P.; Ramlau, R.; Gorbounova, V.; Hirsh, V.; Leighl, N.; Mezger, J.; Archer, V.; Moore, N., et al. Phase III trial of cisplatin plus gemcitabine with either placebo or bevacizumab as first-line therapy for nonsquamous non-small-cell lung cancer: aVAil. *Journal of clinical oncology* **2009**, *27*, 1227-1234, doi:10.1200/JCO.2007.14.5466.
79. Rosell, R.; Carcereny, E.; Gervais, R.; Vergnenegre, A.; Massuti, B.; Felip, E.; Palmero, R.; Garcia-Gomez, R.; Pallares, C.; Sanchez, J.M., et al. Erlotinib versus standard chemotherapy as first-line treatment for European patients with advanced EGFR mutation-positive non-small-cell lung cancer (EURTAC): a multicentre, open-label, randomised phase 3 trial. *The lancet. Oncology* **2012**, *13*, 239-246, doi:10.1016/S1470-2045(11)70393-X.
80. Rosell, R.; Robinet, G.; Szczesna, A.; Ramlau, R.; Constenla, M.; Mennecier, B.C.; Pfeifer, W.; O'Byrne K, J.; Welte, T.; Kolb, R., et al. Randomized phase II study of cetuximab plus cisplatin/vinorelbine compared with cisplatin/vinorelbine alone as first-line therapy in EGFR-expressing advanced non-small-cell lung cancer. *Annals of Oncology* **2008**, *19*, 362-369, doi:10.1093/annonc/mdm474.
81. Saruwatari, K.; Saeki, S.; Sakamoto, Y.; Jodai, T.; Sakata, S.; Tomita, Y.; Nakamura, K.; Hirosako, S.; Okamoto, S.; Ichiyasu, H., et al. Clinical outcomes of epidermal growth factor receptor-mutant non-small cell lung cancer patients with brain metastasis treated with erlotinib versus gefitinib. *American Journal of Respiratory and Critical Care Medicine* **2017**, *195*, doi:10.1164/ajrccm-conference.2017.B80-K.
82. Seto, T.; Kato, T.; Nishio, M.; Goto, K.; Atagi, S.; Hosomi, Y.; Yamamoto, N.; Hida, T.; Maemondo, M.; Nakagawa, K., et al. Erlotinib alone or with bevacizumab as first-line therapy in patients with advanced non-squamous non-small-cell lung cancer harbouring EGFR mutations (JO25567): an open-label,

randomised, multicentre, phase 2 study. *Lancet Oncol* **2014**, *15*, 1236-1244, doi:10.1016/s1470-2045(14)70381-x.
83. Seto, T.; Takeda, M.; Hayashi, H.; Azuma, K.; Okada, M.; Sugawara, S.; Yamanaka, T.; Nakagawa, K.; Nakanishi, Y.; Okamoto, I. Bevacizumab beyond disease progression in advanced non-squamous NSCLC (WJOG 5910L): a randomized phase II trial. *Annals of oncology*. **2015**, *26*, vii91, doi:10.1093/annonc/mdv471.50.
84. Shaw, A.T.; Janne, P.A.; Besse, B.; Solomon, B.J.; Blackhall, F.H.; Camidge, D.R.; Mok, T.; Hirsh, V.; Scranton, J.R.; Polli, A., et al. Crizotinib vs chemotherapy in ALK+ advanced non-small cell lung cancer (NSCLC): Final survival results from PROFILE 1007. *Journal of Clinical Oncology* **2016**, *34*.
85. Shaw, A.T.; Kim, T.M.; Crinò, L.; Gridelli, C.; Kiura, K.; Liu, G.; Novello, S.; Bearz, A.; Gautschi, O.; Mok, T., et al. Ceritinib versus chemotherapy in patients with ALK-rearranged non-small-cell lung cancer previously given chemotherapy and crizotinib (ASCEND-5): a randomised, controlled, open-label, phase 3 trial. *The lancet. Oncology* **2017**, *18*, 874-886, doi:10.1016/S1470-2045(17)30339-X.
86. Soria, J.C.; Tan, D.S.W.; Chiari, R.; Wu, Y.L.; Paz-Ares, L.; Wolf, J.; Geater, S.L.; Orlov, S.; Cortinovis, D.; Yu, C.J., et al. First-line ceritinib versus platinum-based chemotherapy in advanced ALK-rearranged non-small-cell lung cancer (ASCEND-4): a randomised, open-label, phase 3 study. *Lancet* **2017**, *(no pagination)*, doi:10.1016/S0140-6736%2817%2930123-X.
87. Soria, J.C.; Wu, Y.L.; Nakagawa, K.; Kim, S.W.; Yang, J.J.; Ahn, M.J.; Wang, J.; Yang, J.C.; Lu, Y.; Atagi, S., et al. Gefitinib plus chemotherapy versus placebo plus chemotherapy in EGFR-mutation-positive non-small-cell lung cancer after progression on first-line gefitinib (IMPRESS): a phase 3 randomised trial. *The lancet. Oncology* **2015**, *16*, 990-998, doi:10.1016/S1470-2045(15)00121-7.
88. Spigel, D.R.; Anthony Greco, F.; Waterhouse, D.M.; Shipley, D.L.; Zubkus, J.D.; Bury, M.J.; Webb, C.D.; Hart, L.L.; Gian, V.G.; Infante, J.R., et al. Phase II trial of ixabepilone and carboplatin with or without bevacizumab in patients with previously untreated advanced non-small-cell lung cancer. *Lung cancer (Amsterdam, Netherlands)* **2012**, *78*, 70-75, doi:10.1016/j.lungcan.2012.06.008.
89. Spigel, D.R.; Hainsworth, J.D.; Joseph, M.J.; Shipley, D.L.; Hagan, M.K.; Thompson, D.S.; Burris, H.A.; Greco, F.A. Randomized phase 2 trial of pemetrexed, pemetrexed/bevacizumab, and pemetrexed/carboplatin/bevacizumab in patients with stage IIIB/IV non-small cell lung cancer and an Eastern Cooperative Oncology Group performance status of 2. *Cancer* **2018**, *(no pagination)*, doi:10.1002/cncr.30986.
90. Spigel, D.R.; Rubin, M.S.; Gian, V.G.; Shipley, D.L.; Burris, H.A.; Kosloff, R.A.; Shih, K.C.; Quinn, R.; Greco, F.A.; Hainsworth, J.D. Sorafenib and continued erlotinib or sorafenib alone in patients with advanced non-small cell lung cancer progressing on erlotinib: a randomized phase II study of the Sarah Cannon Research Institute (SCRI). *Lung cancer (amsterdam, netherlands)* **2017**, *113*, 79-84, doi:10.1016/j.lungcan.2017.09.007.
91. Stinchcombe, T.E.; Peterman, A.H.; Lee, C.B.; Moore, D.T.; Beaumont, J.L.; Bradford, D.S.; Bakri, K.; Taylor, M.; Crane, J.M.; Schwartz, G., et al. A randomized phase II trial of first-line treatment with gemcitabine, erlotinib, or gemcitabine and erlotinib in elderly patients (age >70 years) with stage IIIB/IV non-small cell lung cancer. *Journal of thoracic oncology* **2011**, *6*, 1569-1577, doi:10.1097/JTO.0b013e3182210430.
92. Sun, J.M.; Lee, K.H.; Kim, S.W.; Lee, D.H.; Min, Y.J.; Yun, H.J.; Kim, H.K.; Song, H.S.; Kim, Y.H.; Kim, B.S., et al. Gefitinib versus pemetrexed as second-line treatment in patients with nonsmall cell lung cancer previously treated with platinum-based chemotherapy (KCSG-LU08-01): an open-label, phase 3 trial. *Cancer* **2012**, *118*, 6234-6242, doi:10.1002/cncr.27630.
93. Takeda, K.; Hida, T.; Sato, T.; Ando, M.; Seto, T.; Satouchi, M.; Ichinose, Y.; Katakami, N.; Yamamoto, N.; Kudoh, S., et al. Randomized phase III trial of platinum-doublet chemotherapy followed by gefitinib compared with continued platinum-doublet chemotherapy in Japanese patients with advanced non-small-cell lung cancer: Results of a West Japan Thoracic Oncology Group Trial (WJTOG0203). *Journal of Clinical Oncology* **2010**, *28*, 753-760, doi:10.1200/JCO.2009.23.3445.
94. Thomas, M.; Fischer, J.; Andreas, S.; Kortsik, C.; Grah, C.; Serke, M.; Von Eiff, M.; Witt, C.; Kollmeier, J.; Muller, E., et al. Erlotinib and bevacizumab versus cisplatin, gemcitabine and bevacizumab in unselected nonsquamous nonsmall cell lung cancer. *European respiratory journal* **2015**, *46*, 219-229, doi:10.1183/09031936.00229014.
95. Thomas, S.; Doebele, R.C.; Spigel, D.; Tehfe, M.; Reck, M.; Verma, S.; Zimmermann, A.; Alexandris, E.; Lee, P.; Bonomi, P. A phase 2 randomized open-label study of ramucirumab (RAM) plus first-line platinum-based chemotherapy in patients (pts) with recurrent or advanced non-small cell lung cancer

(NSCLC): final results from squamous pts. *Annals of oncology. Conference: 7th european lung cancer conference, ELCC 2017. Switzerland* **2017**, *28*, iii42-iii43, doi:10.1093/annonc/mdx085.
96. Urata, Y.; Katakami, N.; Morita, S.; Kaji, R.; Yoshioka, H.; Seto, T.; Satouchi, M.; Iwamoto, Y.; Kanehara, M.; Fujimoto, D., et al. Randomized phase III study comparing gefitinib with erlotinib in patients with previously treated advanced lung adenocarcinoma: WJOG 5108L. *J Clin Oncol* **2016**, *34*, 3248-3257, doi:10.1200/jco.2015.63.4154.
97. Wakelee, H.; Zvirbule, Z.; De Braud, F.; Kingsley, C.D.; Mekhail, T.; Lowe, T.; Schütte, W.; Lena, H.; Lawler, W.; Braiteh, F., et al. Efficacy and safety of onartuzumab in combination with first-line bevacizumab- or pemetrexed-based chemotherapy regimens in advanced non-squamous non-small-cell lung cancer. *Clinical lung cancer* **2017**, *18*, 50-59, doi:10.1016/j.cllc.2016.09.013.
98. Wakelee, H.A.; Gettinger, S.; Engelman, J.; Janne, P.A.; West, H.; Subramaniam, D.S.; Leach, J.; Wax, M.; Yaron, Y.; Miles, D.R., et al. A phase Ib/II study of cabozantinib (XL184) with or without erlotinib in patients with non-small cell lung cancer. *Cancer chemotherapy and pharmacology* **2017**, 10.1007/s00280-017-3283-z, 1-10, doi:10.1007/s00280-017-3283-z.
99. Wang, F.; Ning, F.; Liu, C.; Hao, Y.; Li, L.; Yu, Z.; Chen, S.; Li, B. Comparison of gefitinib versus VMP in the combination with radiotherapy for multiple brain metastases from non-small cell lung cancer. *Cell biochemistry and biophysics* **2015**, *71*, 1261-1265, doi:10.1007/s12013-014-0286-9.
100. Wang, S.Y.; Ou, W.; Li, N.; Sun, H.; Zhang, L.; Fang, Q. Pemetrexed-carboplatin adjuvant chemotherapy with or without gefitinib in resected stage IIIA-N2 non-small cell lung cancer harbouring EGFR mutations: a randomized phase II study. *Journal of clinical oncology* **2013**, *31*.
101. Alliance for Clinical Trials in, O.; National Cancer, I. Cetuximab and radiation therapy in treating patients with stage III non-small cell lung cancer. Availabe online: https://ClinicalTrials.gov/show/NCT00124618
102. Alliance for Clinical Trials in, O.; National Cancer, I.; Eli, L.; Company; Bristol-Myers, S. Pemetrexed disodium, carboplatin, and radiation therapy with or without cetuximab in treating patients with stage III non-small cell lung cancer. Availabe online: https://ClinicalTrials.gov/show/NCT00117962
103. AstraZeneca. First Line IRESSA™ versus carboplatin/paclitaxel in Asia. Availabe online: https://ClinicalTrials.gov/show/NCT00322452
104. AstraZeneca. Assess the efficacy, safety and tolerability of gefitinib (Iressa® 250mg) as maintenance therapy in locally advanced or metastatic (stage IIIB/IV) non small cell lung cancer (NSCLC). Availabe online: https://ClinicalTrials.gov/show/NCT00770588
105. AstraZeneca. A study of IRESSA treatment beyond progression in addition to chemotherapy versus chemotherapy alone. Availabe online: https://ClinicalTrials.gov/show/NCT01544179
106. AstraZeneca. AZD9291 versus platinum-based doublet-chemotherapy in locally advanced or metastatic non-small cell lung cancer. Availabe online: https://ClinicalTrials.gov/show/NCT02151981
107. Boehringer, I. BIBW 2992 (afatinib) versus chemotherapy as first Line treatment in NSCLC with EGFR mutation. Availabe online: https://ClinicalTrials.gov/show/NCT00949650
108. Boehringer, I. LUX-Lung 5: Afatinib plus weekly paclitaxel versus investigator's choice of single agent chemotherapy following afatinib monotherapy in non-small cell lung cancer patients failing erlotinib or gefitinib. Availabe online: https://ClinicalTrials.gov/show/NCT01085136
109. Boehringer, I. LUX-Lung 8: A phase III trial of afatinib (BIBW 2992) versus erlotinib for the treatment of squamous cell lung cancer after at least one prior platinum based chemotherapy. Availabe online: https://ClinicalTrials.gov/show/NCT01523587
110. Boehringer, I. BIBW 2992 (afatinib) vs gemcitabine-cisplatin in 1st line non-small cell lung cancer (NSCLC). Availabe online: https://ClinicalTrials.gov/show/NCT01121393
111. Boehringer, I. BIBW 2992 and BSC versus placebo and BSC in non-small cell lung cancer patients failing erlotinib or gefitinib (LUX-LUNG 1). Availabe online: https://ClinicalTrials.gov/show/NCT00656136
112. Case Comprehensive Cancer, C.; National Cancer, I. Pemetrexed or docetaxel with or without erlotinib in stage IIIB or stage IV non-small cell lung cancer. Availabe online: https://ClinicalTrials.gov/show/NCT00660816
113. Center, H.L.M.C.; Research, I.; National Cancer, I.; AstraZeneca. Neoadjuvant IRESSA as single agent preoptherapy for NSCLC with molecular correlates. Availabe online: https://ClinicalTrials.gov/show/NCT00104728
114. Center, M.D.A.C. Tarceva surgery for resectable stage IIIA(N2) and IIIB (T4 N2) non-small-cell lung cancer. Availabe online: https://ClinicalTrials.gov/show/NCT00063258

115. Center, U.N.C.L.C.C.; National Cancer, I. Ph II gemcitabine, erlotinib, and gemcitabine with erlotinib/elderly patients W/ IIIB/IV NSCLC. Availabe online: https://ClinicalTrials.gov/show/NCT00283244
116. Eastern Cooperative Oncology, G.; National Cancer, I. Cetuximab in treating patients with recurrent or stage IIIB or stage IV lung cancer. Availabe online: https://ClinicalTrials.gov/show/NCT00103207
117. Eli, L.; Company. Chemotherapy for patients with non-small cell lung cancer who are non-smokers. Availabe online: https://ClinicalTrials.gov/show/NCT00409006
118. Eli, L.; Company. Study of pemetrexed versus pemetrexed plus erlotinib as treatment of nonsquamous non-small cell lung cancer (NSCLC). Availabe online: https://ClinicalTrials.gov/show/NCT00447057
119. Eli, L.; Company. A study for non-smoker patients with nonsquamous non-small cell lung cancer. Availabe online: https://ClinicalTrials.gov/show/NCT00550173
120. Eli, L.; Company. Study of participants with advanced non-small cell lung cancer. Availabe online: https://ClinicalTrials.gov/show/NCT00948675
121. Eli, L.; Company. A study of docetaxel and ramucirumab versus docetaxel and placebo in the treatment of stage IV non-small cell lung cancer. Availabe online: https://ClinicalTrials.gov/show/NCT01703091
122. Eli, L.; Company. A study of pemetrexed and carboplatin/cisplatin or gemcitabine and carboplatin/cisplatin with or without IMC-1121B in participants previously untreated with recurrent or advanced non-small cell lung cancer (NSCLC). Availabe online: https://ClinicalTrials.gov/show/NCT01160744
123. Eli, L.; Company; ImClone, L.L.C. Study of taxane/carboplatin +/- cetuximab as first-line treatment for patients with advanced/metastatic non-small cell lung cancer. Availabe online: https://ClinicalTrials.gov/show/NCT00112294
124. Eli, L.; Company; ImClone, L.L.C. A study of cetuximab and bevacizumab in combination with paclitaxel and carboplatin in stage IIIb/IV NSCLC. Availabe online: https://ClinicalTrials.gov/show/NCT00343291
125. Eli, L.; Company; ImClone, L.L.C. Docetaxel or pemetrexed with or without cetuximab in patients with recurrent or progressive non-small cell lung cancer. Availabe online: https://ClinicalTrials.gov/show/NCT00095199
126. Eli, L.; Company; ImClone, L.L.C. A study of chemotherapy and ramucirumab versus chemotherapy alone in second line non-small cell lung cancer (NSCLC) participants who received prior first line platinum-based chemotherapy. Availabe online: https://ClinicalTrials.gov/show/NCT01168973
127. European Organisation for, R.; Treatment of Cancer, E. Gefitinib after chemotherapy in treating patients with stage IIIB or stage IV non-small cell lung cancer. Availabe online: https://ClinicalTrials.gov/show/NCT00091156
128. Exelixis. A study of XL184 (cabozantinib) with or without erlotinib in adults with non-small cell lung cancer. Availabe online: https://ClinicalTrials.gov/show/NCT00596648
129. Gachon University Gil Medical, C. Gefitinib versus pemetrexed for previously treated NSCLC patients. Availabe online: https://ClinicalTrials.gov/show/NCT01783834
130. Genentech, I. A study to evaluate the efficacy of bevacizumab in combination with tarceva for advanced non-small cell lung cancer. Availabe online: https://ClinicalTrials.gov/show/NCT00130728
131. Genentech, I. A study comparing bevacizumab therapy with or without erlotinib for first-line treatment of non-small cell lung cancer (ATLAS). Availabe online: https://ClinicalTrials.gov/show/NCT00257608
132. Genzyme, a.S.C.; Sanofi. ZACTIMA (an anti-EGFR anti-VEGF agent) combined with docetaxel compared to docetaxel in non-small cell lung cancer. Availabe online: https://ClinicalTrials.gov/show/NCT00312377
133. Genzyme, a.S.C.; Sanofi. Efficacy study comparing ZD6474 in combination with pemetrexed and pemetrexed alone in 2nd line NSCLC patients. Availabe online: https://ClinicalTrials.gov/show/NCT00418886
134. Genzyme, a.S.C.; Sanofi. Efficacy trial comparing ZD6474 with erlotinib in NSCLC after failure of at least one prior chemotherapy. Availabe online: https://ClinicalTrials.gov/show/NCT00364351
135. Genzyme, a.S.C.; Sanofi. ZD6474 (ZACTIMA™) phase III study in EGFR failures. Availabe online: https://ClinicalTrials.gov/show/NCT00404924
136. Genzyme, a.S.C.; Sanofi. Phase II of Zactima maintenance for locally advanced or metastatic non-small-cell lung carcinoma (NSCLC) following platinum-doublet chemotherapy. Availabe online: https://ClinicalTrials.gov/show/NCT00777179
137. Genzyme, a.S.C.; Sanofi. Zactima in non small cell lung cancer (NSCLC) eLderly patients in combination with or versus gemcitabine. Availabe online: https://ClinicalTrials.gov/show/NCT00753714

138. Hoffmann-La, R. A Study of Tarceva (erlotinib) and gemcitabine in treatment-naive patients with advanced non-small cell lung cancer. Availabe online: https://ClinicalTrials.gov/show/NCT00518011
139. Hoffmann-La, R. A Study of Tarceva (erlotinib) following platinum-based chemotherapy in patients with advanced, recurrent, or metastatic non-small cell lung cancer (NSCLC). Availabe online: https://ClinicalTrials.gov/show/NCT00556712
140. Hoffmann-La, R. A study of Tarceva (erlotinib) in combination with Avastin (bevacizumab) in patients with advanced non-small cell lung cancer. Availabe online: https://ClinicalTrials.gov/show/NCT00531960
141. Hoffmann-La, R. A study of Tarceva (erlotinib) in elderly patients with advanced non-small cell lung cancer. Availabe online: https://ClinicalTrials.gov/show/NCT01196078
142. Hoffmann-La, R. A Study of Tarceva (erlotinib) in combination with platinum based chemotherapy in patients with non-small cell lung cancer. Availabe online: https://ClinicalTrials.gov/show/NCT01998919
143. Hoffmann-La, R. A study of Avastin (bevacizumab) in patients with non-squamous non-small cell lung cancer with asymptomatic untreated brain metastasis. Availabe online: https://ClinicalTrials.gov/show/NCT00800202
144. Hoffmann-La, R. A study of erlotinib (Tarceva) versus gemcitabine/cisplatin as first-line treatment in patients with non-small cell lung cancer with EGFR mutations. Availabe online: https://ClinicalTrials.gov/show/NCT01342965
145. Hoffmann-La, R. A Study of Tarceva (erlotinib) and standard of care chemotherapy in patients with advanced, recurrent, or metastatic non-small cell lung cancer (NSCLC). Availabe online: https://ClinicalTrials.gov/show/NCT00556322
146. Hoffmann-La, R. A study of Tarceva (erlotinib) or placebo in combination with platinum-based therapy as first line treatment in patients with advanced or recurrent non-small cell lung cancer. Availabe online: https://ClinicalTrials.gov/show/NCT00883779
147. Hoffmann-La, R. A study comparing alectinib with crizotinib in treatment-naive anaplastic lymphoma kinase-positive advanced aon-small cell lung cancer participants. Availabe online: https://ClinicalTrials.gov/show/NCT02075840
148. Hoffmann-La, R. Alectinib versus pemetrexed or docetaxel in Anaplastic Lymphoma Kinase (ALK)-positive advanced non-small cell lung cancer (NSCLC) participants previously treated with platinum-based chemotherapy and crizotinib. Availabe online: https://ClinicalTrials.gov/show/NCT02604342
149. Memorial Sloan Kettering Cancer, C.; National Cancer, I. Gefitinib and everolimus in treating patients with stage IIIB or stage IV or Recurrent Non-Small Cell Lung Cancer. Availabe online: https://ClinicalTrials.gov/show/NCT00096486
150. Merck, K. Study of cisplatin/vinorelbine +/- cetuximab as first-line treatment of advanced non small cell lung cancer (FLEX). Availabe online: https://ClinicalTrials.gov/show/NCT00148798
151. National Cancer Center, K.; AstraZeneca. Intercalated administration of PamCis with gefitinib or placebo as first line lung adenocarcinoma in never smokers. Availabe online: https://ClinicalTrials.gov/show/NCT01502202
152. National Cancer, I. Erlotinib hydrochloride with or without carboplatin and paclitaxel in treating patients with stage III-IV non-small cell lung cancer. Availabe online: https://ClinicalTrials.gov/show/NCT00126581
153. National Cancer, I. Gefitinib in treating patients with stage IB, II, or IIIA non-small cell lung cancer that was completely removed by surgery. Availabe online: https://ClinicalTrials.gov/show/NCT00049543
154. National Cancer, I. Erlotinib hydrochloride and cabozantinib-s-malate alone or in combination as second or third line therapy in treating patients with stage IV non-small cell lung cancer. Availabe online: https://ClinicalTrials.gov/show/NCT01708954
155. National Cancer, I.; Radiation Therapy Oncology, G. Radiation therapy and stereotactic radiosurgery with or without temozolomide or erlotinib in treating patients with brain metastases secondary to non-small cell lung cancer. Availabe online: https://ClinicalTrials.gov/show/NCT00096265
156. Novartis, P.; Novartis. LDK378 versus chemotherapy in ALK rearranged (ALK positive) patients previously treated with chemotherapy (platinum doublet) and crizotinib. Availabe online: https://ClinicalTrials.gov/show/NCT01828112
157. Novartis, P.; Novartis. LDK378 versus chemotherapy in previously untreated patients with ALK rearranged non-small cell lung cancer. Availabe online: https://ClinicalTrials.gov/show/NCT01828099
158. Pfizer. An investigational drug, PF-02341066 is being studied versus standard of care in patients with advanced non-small cell lung cancer with a specific gene profile involving the anaplastic lymphoma kinase (ALK) gene. Availabe online: https://ClinicalTrials.gov/show/NCT00932893

159. Pfizer. A clinical trial testing the efficacy of crizotinib versus standard chemotherapy pemetrexed plus cisplatin or carboplatin in patients with ALK positive non squamous cancer of the lung. Availabe online: https://ClinicalTrials.gov/show/NCT01154140
160. Pfizer. A study of crizotinib versus chemotherapy in previously untreated ALK positive East Asian non-small cell lung cancer patients. Availabe online: https://ClinicalTrials.gov/show/NCT01639001
161. Pharmaceuticals, O.S.I.; Astellas Pharma, I. Erlotinib vs. standard chemotherapy in patients with advanced non-small cell lung cancer (NSCLC) and Eastern Cooperative Oncology Group (ECOG) performance status (PS) 2. Availabe online: https://ClinicalTrials.gov/show/NCT00085839
162. Pharmaceuticals, O.S.I.; Astellas Pharma, I. A study of erlotinib (Tarceva) after surgery with or without adjuvant chemotherapy in non-small cell lung carcinoma (NSCLC) patients who have epidermal growth factor receptor (EGFR) positive tumors. Availabe online: https://ClinicalTrials.gov/show/NCT00373425
163. PrEcog, L.L.C.; AstraZeneca. Study of vandetanib combined with chemotherapy to treat advanced non-small cell lung cancer. Availabe online: https://ClinicalTrials.gov/show/NCT00687297
164. Scri Development Innovations, L.L.C.; Bayer; Pharmaceuticals, O.S.I. Sorafenib and erlotinib or sorafenib alone in advanced non-small cell lung cancer progressing on Erlotinib. Availabe online: https://ClinicalTrials.gov/show/NCT00609804
165. Scri Development Innovations, L.L.C.; Bristol-Myers, S.; Genentech, I. Ixabepilone and carboplatin +/- bevacizumab in advanced non-small-cell lung cancer. Availabe online: https://ClinicalTrials.gov/show/NCT00741988
166. Scri Development Innovations, L.L.C.; Genentech, I. Trial of poor performance status patients (ToPPS). Availabe online: https://ClinicalTrials.gov/show/NCT00892710
167. Si-Yu, W.; Sun Yat-sen, U. Erlotinib versus pemetrexed as second-line therapy in treating patients with advanced lung adenocarcinoma. Availabe online: https://ClinicalTrials.gov/show/NCT01565538
168. Southwest Oncology, G.; National Cancer, I. S0819: Carboplatin and paclitaxel with or without bevacizumab and/or cetuximab in treating patients with stage IV or recurrent non-small cell lung cancer. Availabe online: https://ClinicalTrials.gov/show/NCT00946712
169. Spectrum Pharmaceuticals, I. Study of pralatrexate vs. erlotinib for non-small cell lung cancer after at least 1 prior platinum-based treatment. Availabe online: https://ClinicalTrials.gov/show/NCT00606502
170. University of, C.; Bristol-Myers, S. Trial comparing cetuximab with pemetrexed/cetuximab therapy for non-small cell lung cancer. Availabe online: https://ClinicalTrials.gov/show/NCT00203931
